# Integration of pre-treatment computational radiomics, deep radiomics, and transcriptomics enhances soft-tissue sarcoma patient prognosis

**DOI:** 10.1038/s41698-024-00616-8

**Published:** 2024-06-07

**Authors:** Amandine Crombé, Carlo Lucchesi, Frédéric Bertolo, Michèle Kind, Mariella Spalato-Ceruso, Maud Toulmonde, Vanessa Chaire, Audrey Michot, Jean-Michel Coindre, Raul Perret, François Le Loarer, Aurélien Bourdon, Antoine Italiano

**Affiliations:** 1 Department of Oncologic Imaging, Bergonié Institute, F-33076 Bordeaux, France; 2 Department of Radiology, Pellegrin University Hospital, F-33076 Bordeaux, France; 3 Bordeaux Institute of Oncology, BRIC U1312, Sarcotarget team, INSERM, University of Bordeaux, Institut Bergonié, F-33000 Bordeaux, France; 4 Department of Bioinformatics, Bergonié Institute, F-33076 Bordeaux, France; 5 Department of Medical Oncology, Bergonié Institute, F-33076 Bordeaux, France; 6 Department of Pathology, Bergonié Institute, F-33076 Bordeaux, France; 7 Department of Oncologic Surgery, Bergonié Institute, F-33076 Bordeaux, France

**Keywords:** Sarcoma, Cancer imaging, Prognostic markers

## Abstract

Our objective was to capture subgroups of soft-tissue sarcoma (STS) using handcraft and deep radiomics approaches to understand their relationship with histopathology, gene-expression profiles, and metastatic relapse-free survival (MFS). We included all consecutive adults with newly diagnosed locally advanced STS (*N* = 225, 120 men, median age: 62 years) managed at our sarcoma reference center between 2008 and 2020, with contrast-enhanced baseline MRI. After MRI postprocessing, segmentation, and reproducibility assessment, 175 handcrafted radiomics features (h-RFs) were calculated. Convolutional autoencoder neural network (CAE) and half-supervised CAE (HSCAE) were trained in repeated cross-validation on representative contrast-enhanced slices to extract 1024 deep radiomics features (d-RFs). Gene-expression levels were calculated following RNA sequencing (RNAseq) of 110 untreated samples from the same cohort. Unsupervised classifications based on h-RFs, CAE, HSCAE, and RNAseq were built. The h-RFs, CAE, and HSCAE grouping were not associated with the transcriptomics groups but with prognostic radiological features known to correlate with lower survivals and higher grade and SARCULATOR groups (a validated prognostic clinical-histological nomogram). HSCAE and h-RF groups were also associated with MFS in multivariable Cox regressions. Combining HSCAE and transcriptomics groups significantly improved the prognostic performances compared to each group alone, according to the concordance index. The combined radiomic-transcriptomic group with worse MFS was characterized by the up-regulation of 707 genes and 292 genesets related to inflammation, hypoxia, apoptosis, and cell differentiation. Overall, subgroups of STS identified on pre-treatment MRI using handcrafted and deep radiomics were associated with meaningful clinical, histological, and radiological characteristics, and could strengthen the prognostic value of transcriptomics signatures.

## Introduction

Soft-tissue sarcomas (STS) represent prevalent malignant mesenchymal tumors characterized by diverse clinical and radiological presentations, along with distinctive histologic and molecular features, influencing their prognosis^1^. Contrast-enhanced (CE) MRI stands out as the optimal imaging modality for local staging of locally advanced STS, revealing various radiological phenotypes (radiophenotypes) and substantial intra- and inter-tumoral heterogeneity within and between STS^2^. A qualitative assessment of these radiophenotypes using conventional MRI sequences has been correlated with the French Federation of Cancer Center (FNCLCC) histologic grading, metastasis-free survival (MFS) and overall survival (OS)^3,4^, notably peritumoral enhancement after Gadolinium chelates injection, necrotic signal and marked heterogeneous signal intensity on T2-weigted imaging. However, this assessment remains subjective, inadequately reproducible, and incapable of capturing the intricate intra-tumoral patterns present in STS.

Radiomics entails the extensive quantification of the radiophenotype in medical imaging from any modality. It employs mathematical operators to derive numeric data, referred to as radiomics features (RFs), capturing aspects of tumor shape and texture, including three-dimensional rearrangements of gray levels within the tumor^5^. These RFs are subsequently explored in supervised machine-learning algorithms for predicting various oncologic outcomes or in unsupervised clustering algorithms to unveil hidden patterns within the data. The underlying hypothesis is that radiomics, on a macroscopic scale, reflects the molecular features of cancers, potentially serving as a non-invasive virtual biopsy^6^. Previous studies have successfully trained radiomics models to predict FNCLCC grade^7,8^, response to neoadjuvant chemotherapy or radiotherapy^9–11^, and patient survival^12–14^. However, only one exploratory study has directly correlated gene-expression profiles of STS with radiomics on a subset of 21 patients, suggesting links between radiomics clusters and pathways involved in apoptosis, immune infiltrates, and cell proliferation^15^. Conversely, predictive gene-expression signatures dedicated to STS have never been put in perspective with conventional radiological features or radiomics data^16^.

Recently, handcrafted RFs (h-RFs) have been complemented with deep RFs (d-RFs) obtained from the last fully connected layer of pre-trained convolutional neural networks. They aim to refine and personalize radiophenotypic quantification, though preliminary findings and concerns about their lack of explainability exist^17,18^. In particular, convolutional auto-encoder neural networks (CAE) are powerful deep learning techniques that extract the most meaningful features from medical images through encoder–decoder architecture, which compresses medical images into a low dimensional space and then reconstruct them as accurately as possible after retaining the most relevant characteristics (i.e., the d-RFs). Although these networks can remain completely unsupervised, it is also possible to provide output labels to each image to guide the learning process towards specific objectives, such as prognostication in half-supervised CAE (HSCAE). Comparing CAE and HSCAE can help in understanding the benefits of including labeled data (in terms of better image reconstruction, feature extraction, or better classification) and be valuable to estimate the intrinsic information contained in medical images. An objective and larger assessment of the added value and potentiation of h-RF, d-RF (either CAE or HSCAE), and transcriptomics data in STS patients is currently lacking. Additionally, understanding their relationships with semantic radiological features^3,4^ (i.e., explainable with medical language by radiologists) and the clinical–histological SARCULATOR nomogram of reference would facilitate the acceptance of these complex ‘-omics’ data^19^.

Therefore, the overarching objective of this study was to conduct an exploratory and comprehensive assessment of the inter-relations and potentialities of multi-omics data in newly diagnosed, locally advanced STS without preconceived notions. Our specific aims were (i) to identify patterns of STS using h-RFs, d-RFs, and transcriptomics data, (ii) to investigate the associations of the corresponding h-RF, d-RF, and transcriptomics groups among themselves with the SARCULATOR predictions, and with semantic radiological features known to correlate with grade and metastatic relapse-free survival (MFS), and (iii) to explore their prognostic value alone and in combination.

## Results

### Patient characteristics

**Fig. 1:**
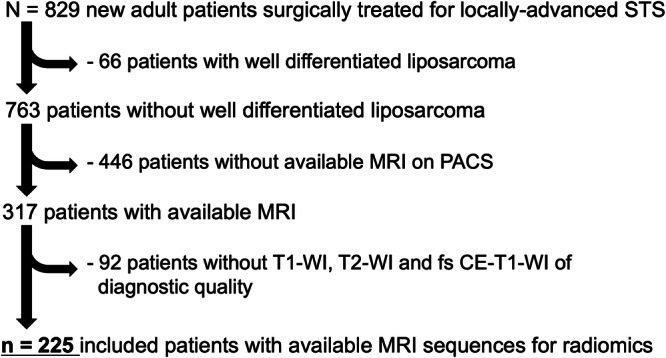
Study flow chart. fs fat suppressed, PACS picture archiving and communication system, STS soft tissue sarcoma, WI weighted imaging.

The study flowchart is shown in Fig. 1. Briefly, out of 829 patients initially identified, 225 were included (median age: 61.2 years, range: 18–95, 120/225 [53.3%] men). Characteristics are presented in Table 1, with the most prevalent histotype being undifferentiated pleomorphic sarcoma (UPS, 71/225 [31.6%]). The majority of STS were strictly deep-seated (136/225 [70.4%]), located in the lower limbs (126/225 [56%]), and had a high (III) histologic grade (116/222 [52.3%], with 3 patients having non-available grade). One hundred and ten patients had sufficient materials for the transcriptomics analysis, whose characteristics are also shown in Table 1.

**Table 1:** Patients’ characteristics

Characteristics	Cohorts				*P*-value training vs. testing^a^
	All (*N* = 225)	Patients with RNAseq material (*n* = 110)	Training (*n* = 200)	Testing (*n* = 25)	
*Age*					
Mean ± SD	59.5 ± 16.4	58.2 ± 18.3	59.6 ± 16.8	58.5 ± 12.4	0.5810
Median (range)	62 (18–95)	60 (18–90)	61.5 (18– 95)	62 (27– 76)	
*Sex*					
Men	120/225 (53.3)	61/110 (55.5)	106/200 (53)	14/25 (56)	0.9435
Women	105/225 (46.7)	49/110 (44.5)	94/200 (47)	11/25 (44)	
*WHO performance status*					
0	180/225 (80)	89/110 (80.9)	163/200 (81.5)	17/25 (68)	0.2395
≥1	45/225 (20)	21/110 (19.1)	37/200 (18.5)	8/25 (32)	
*Histologic type*					
Angiosarcoma	1/225 (0.4)	0/110 (0)	1/200 (0.5)	0/25 (0)	0.5524
Extraskeletal myxoid chondrosarcoma	6/225 (2.7)	4/110 (3.6)	6/200 (3)	0/25 (0)	
Dedifferentiated LPS	15/225 (6.7)	12/110 (10.9)	15/200 (7.5)	0/25 (0)	
Leiomyosarcoma	24/225 (10.7)	12/110 (10.9)	22/200 (11)	2/25 (8)	
Myxofibrosarcoma	13/225 (5.8)	8/110 (7.3)	13/200 (6.5)	0/25 (0)	
MPNST	3/225 (1.3)	3/110 (2.7)	3/200 (1.5)	0/25 (0)	
Myxoid/round cells LPS	22/225 (9.8)	8/110 (7.3)	18/200 (9)	4/25 (16)	
Pleomorphic LPS	12/225 (5.3)	6/110 (5.5)	11/200 (5.5)	1/25 (4)	
Rhabdomyosarcoma	13/225 (5.8)	6/110 (5.5)	11/200 (5.5)	2/25 (8)	
Sarcoma NOS	9/225 (4)	3/110 (2.7)	9/200 (4.5)	0/25 (0)	
Low-grade fibromyxoid sarcoma	4/225 (1.8)	4/110 (3.6)	4/200 (2)	0/25 (0)	
Solitary fibrous tumor	7/225 (3.1)	3/110 (2.7)	7/200 (3.5)	0/25 (0)	
Synovial sarcoma	16/225 (7.1)	8/110 (7.3)	13/200 (6.5)	3/25 (12)	
UPS	71/225 (31.6)	29/110 (26.4)	59/200 (29.5)	12/25 (48)	
US other	5/225 (2.2)	2/110 (1.8)	5/200 (2.5)	0/25 (0)	
Other	4/225 (1.8)	2/110 (1.8)	3/200 (1.5)	1/25 (4)	
*Size*					
Mean ± SD	98.4 ± 52.3	94.7 ± 50.9	95.4 ± 50.7	122.8 ± 60	0.0350*
Median (range)	87 (19– 289)	81 (19–260)	85.5 (19– 289)	112 (52– 247)	
*Depth*					
Superficial	9/225 (4)	7/110 (6.4)	9/200 (4.5)	0/25 (0)	0.1914
Deep	136/225 (60.4)	59/110 (53.6)	117/200 (58.5)	19/25 (76)	
In between’	80/225 (35.6)	44/110 (40)	74/200 (37)	6/25 (24)	
*Location*					
Lower limb	126/225 (56)	63/110 (57.3)	107/200 (53.5)	19/25 (76)	0.06115
Girdles	34/225 (15.1)	16/110 (14.5)	30/200 (15)	4/25 (16)	
Trunk	39/225 (17.3)	21/110 (19.1)	39/200 (19.5)	0/25 (0)	
Upper limb	26/225 (11.6)	10/110 (9.1)	24/200 (12)	2/25 (8)	
*FNCLCC grade*^b^					
I	30/222 (13.5)	17/108 (15.7)	30/197 (15.2)	0/25 (0)	0.09692
II	76/222 (34.2)	36/108 (33.3)	65/197 (33)	11/25 (44)	
III	116/222 (52.3)	55/108 (50.9)	102/197 (51.8)	14/25 (56)	
*Radiotherapy*					
No	31/225 (13.8)	16/110 (14.5)	30/200 (15)	1/25 (4)	0.2314
Yes	194/225 (86.2)	94/110 (85.5)	170/200 (85)	24/25 (96)	
*Chemotherapy*					
No	101/225 (44.9)	69/110 (62.7)	101/200 (50.5)	0/25 (0)	<0.0001***
Yes	124/225 (55.1)	41/110 (37.3)	99/200 (49.5)	25/25 (100)	
*Surgical margins*					
R0	144/221 (65.2)	78/110 (70.9)	130/200 (65)	14/21 (66.7)	0.9415
R1	76/221 (34.4)	32/110 (29.1)	69/200 (34.5)	7/21 (33.3)	
R2	1/221 (0.5)	0/110 (0)	1/200 (0.5)	0/25 (0)	

Data are a number of patients with percentages in parentheses, except for age and tumor size.

*FNCLCC* French Federation of Cancer Centers, *LPS* liposarcoma, *MPNST* malignant peripheral nerve sheath tumor, *NOS* not otherwise specified, *SD* standard deviation, *UPS* US, *WHO* World Health Organization.

^a^*P*-values correspond to the Chi-square tests. *P < 0.05, ***P < 0.001. Significant results are in bold.

^b^Grade was assessed on imaging-guided biopsy for 118/222 (53.2%) patients, on whole surgical specimens for 104/222 (46.8%) patients, and nonanalyzable for 3 patients.

### Comprehensive patient clustering

Unsupervised consensus clustering on h-RFs identified three groups: A_h-RF_ (71/225, 31.6%), B_h-RF_ (87/225, 38.7%), and C_h-RF_ (67/225, 28.9%).

Regarding the development of the CAE and HSCAE neural networks for the extraction of d-RFs, the Training and Testing cohorts only differed regarding tumor size (*P* = 0.0350) and the number of patients who underwent chemotherapy in addition to surgery (*P* < 0.0001) (Table 1). After training the two convolutional neural network models, we verified that the image reconstruction error according to mean square error (MSE) remains below 1% in both Training and Testing cohorts (Supplementary Table ST1). The unsupervised clustering developed in the HSCAE and CAE d-RFs from the Training cohort systematically provided two groups, named A and B, with cluster A (i.e., A_CAE_ and A_HSCAE_) for each classification referring to the most numerous group. We then applied the centroid assignment technique to label the observations from the Testing cohort.

Unsupervised Consensus clustering on the transcriptomics data identified two groups: A_RNA_ (69/110, 62.7%) and B_RNA_ (41/110, 37.3%), which were significantly associated with the histological type (*P* < 0.0001).

### Understanding patient clustering

Significant associations between radiomics-based clustering and clinical, histological, radiological assessment, SARCULATOR groups, and transcriptomics clustering were observed (Table 2). Radiomics clusters were systematically associated with size, SARCULATOR groups, and semantic radiophenotypes (all *P*-values < 0.001). Moreover, the d-RF clusters (using both the CAE and HSCAE approaches) were consistently associated with FNCLCC grade (*P*-value range: 0.0168–0.0274), while h-RFs groups were not (*P* = 0.1559). Thus, the B_h-RF_ corresponded to STS with the largest size (131 ± 52 mm versus 96 ± 39 mm for A_h-RF_ and 59 ± 33 mm for C_h-RF_—*P* < 0.0001), the higher rate of low Pr-OS STS (42.5% versus 22.5% for A_h-RF_ and 12.5% for B_h-RF_—*P* = 0.0001), and high-risk semantic radiophenotype (71.3% versus 53.5% for A_h-RF_ and 38.8% for C_h-RF_, *P* = 0.0005). Regarding the deep-radiomics clusters, the same trend towards more aggressive presentations was observed for the STS in the A_CAE_ and A_HSCAE_ groups compared to B_CAE_ and B_HSCAE_ groups, respectively (i.e., larger size, lower Pr-OS, and higher rates of high-risk semantic radiophenotype). Moreover, there were significantly more FNCLCC grade III STS in A_CAE_ and A_HSCAE_ groups (58.7% and 58.8%, respectively) compared to the B_CAE_ and B_HSCAE_ groups (41.7% [*P* = 0.0274] and 41.9% [*P* = 0.0168], respectively).

**Table 2:** Associations with the radiomics-based clustering

	h-RFs				CAE			HSCAE		
	A_h-RFS_	B_h-RFS_	C_h-RFs_	*P*-value^a^	A_CAE_	B_CAE_	*P*-value^a^	A _HSCAE_	B _HSCAE_	*P*-value^a^
*Size (mm)*				<0.0001***			<0.0001***			<0.0001***
Mean ± SD	96 ± 39	131 ± 52	59 ± 33		120 ± 51	64 ± 34		121 ± 50	64 ± 35	
*Histotype*				0.0101*			0.2498			0.2634
M/RC LPS	7/71 (9.9)	13/87 (14.9)	2/67 (3)		16/138 (11.6)	6/87 (6.9)		15/136 (11)	7/89 (7.9)	
DD/Pleomorphic LPS	5/71 (7)	16/87 (18.4)	6/67 (9)		19/138 (13.8)	8/87 (9.2)		20/136 (14.7)	7/89 (7.9)	
Leiomyosarcoma	4/71 (5.6)	10/87 (11.5)	10/67 (14.9)		13/138 (9.4)	11/87 (12.6)		11/136 (8.1)	13/89 (14.6)	
Myxofiborsarcoma	2/71 (2.8)	4/87 (4.6)	7/67 (10.4)		4/138 (2.9)	9/87 (10.3)		5/136 (3.7)	8/89 (9)	
Other	19/71 (26.8)	11/87 (12.6)	22/67 (32.8)		30/138 (21.7)	22/87 (25.3)		30/136 (22.1)	22/89 (24.7)	
Synovial sarcoma	6/71 (8.5)	6/87 (6.9)	4/67 (6)		10/138 (7.2)	6/87 (6.9)		9/136 (6.6)	7/89 (7.9)	
UPS	28/71 (39.4)	27/87 (31)	16/67 (23.9)		46/138 (33.3)	25/87 (28.7)		46/136 (33.8)	25/89 (28.1)	
*FNCLCC grade*				0.1559			0.0274*			0.0168*
Grade I	12/71 (16.9)	7/87 (8)	11/64 (17.2)		13/138 (9.4)	17/84 (20.2)		12/136 (8.8)	18/86 (20.9)	
Grade II	29/71 (40.8)	27/87 (31)	20/64 (31.2)		44/138 (31.9)	32/84 (38.1)		44/136 (32.4)	32/86 (37.2)	
Grade III	30/71 (42.3)	53/87 (60.9)	33/64 (51.6)		81/138 (58.7)	35/84 (41.7)		80/136 (58.8)	36/86 (41.9)	
*SARCULATOR groups*				0.0001***			<0.0001***			<0.0001***
High Pr-OS	38/71 (53.5)	25/87 (28.7)	42/64 (65.6)		47/138 (34.1)	58/84 (69)		44/136 (32.4)	61/86 (70.9)	
Interm. Pr-OS	17/71 (23.9)	25/87 (28.7)	14/64 (21.9)		37/138 (26.8)	19/84 (22.6)		37/136 (27.2)	19/86 (22.1)	
Low Pr-OS	16/71 (22.5)	37/87 (42.5)	8/64 (12.5)		54/138 (39.1)	7/84 (8.3)		55/136 (40.4)	6/86 (7)	
*Semantic radiophenotypes*				0.0005***			<0.0001***			<0.0001***
Low risk	33/71 (46.5)	25/87 (28.7)	41/67 (61.2)		39/138 (28.3)	60/87 (69)		42/136 (30.9)	57/89 (64)	
High risk	38/71 (53.5)	62/87 (71.3)	26/67 (38.8)		99/138 (71.7)	27/87 (31)		94/136 (69.1)	32/89 (36)	
*Transcriptomics groups*				0.1867			0.9942			0.9129
B_RNA_	14/38 (36.8)	18/38 (47.4)	9/34 (26.5)		24/63 (38.1)	17/47 (36.2)		25/65 (38.5)	16/45 (35.6)	
A_RNA_	24/38 (63.2)	20/38 (52.6)	25/34 (73.5)		39/63 (61.9)	30/47 (63.8)		40/65 (61.5)	29/45 (64.4)	

Data are number of patients with percentage parentheses.

*CAE* convolutional autoencoder neural network, *DD* dedifferentiated, *FNCLCC* French federation of cancer centers, *h-RFs* handcrafted radiomics features, *HSCAE* half supervised convolutional autoencoder neural network, *LPS* liposarcoma, *M/RC* myxoid/round cells, *Pr-OS* predicted overall survival, *SD* standard deviation, *UPS* undifferentiated pleomorphic sarcoma.

*P < 0.05, ***P < 0.001. Significant results are in bold.

^a^Tests are Chi-square tests for pairs of categorical variables, and, regarding association with size, unpaired Mann–Whitney tests (for CAE and HSCAE) and Friedman test (for h-RFs).

The h-RF clustering was also significantly associated with the histological type (*P* = 0.0101), with a higher proportion of UPS in the A_h-RF_ group (28/71, 39.4%) compared to the C_h-RF_ group (16/67, 23.9%). This weak but significant association between imaging patterns and histological types was also observed with the semantic radiophenotypes (*P* = 0.0005—Chi-square test), again, with higher rates of UPS in the high-risk group (53/126, 42.1%) compared to the low-risk group (18/99, 18.2%).

However, none of the radiomics groups showed an association with the transcriptomics groups (*P*-value range: 0.1867–0.9942), but there were strong associations within themselves in pairwise comparisons (all *P*-values < 0.0001). In particular, the CAE and HSCAE grouping were strongly concordant, except for 18/225 (8%) patients, including 10 patients in the A_CAE_ but B_HSCAE_ groups and 8 patients in the B_CAE_ but A_HSCAE_ groups.

Lastly, the transcriptomics groups were significantly associated with the histological types (*P* < 0.0001) and the FNCLCC grade, with higher rates of grade III STS in the A_RNA_ group compared to the B_RNA_ group (44/67 [65.7%] versus 11/41 [26.8%]—*P* < 0.0001, 2 out of 110 patients without available grade).

### Prognostic value of radiomics groups

Metastatic relapses occurred in 70/225 (31.1%) patients. MFS probability at 2 and 5 years was 78.9% (95%CI: 73.7–84.5) and 66.4% (95%CI: 59.8–73.7), respectively.

Survival analyses for each radiomics cluster (Table 3) revealed worse MFS in univariable analysis, with B_h-RF_ and A_HSCAE_ groups remaining associated in multivariable analysis (univariable analyses for covariables are provided in Supplementary Table ST2).

**Table 3:** Survival analysis for metastatic relapse-free survival

Characteristics	No. at risk	No. of events	5-year MFS probability	Log-rank *P*-value	Univariable HR (95% CI)	*P*-value	Multivariable HR (95%CI)^a^	*P*-value
*h-Radiomics groups*				0.0081*				
C_h-RF_ (ref.)	67	12	83 (73– 93)		*reference*	–	*reference*	–
A_h-RF_	71	24	62 (51– 77)		2.11 (1.05–4.23)	0.0349*	1.97 (0.90– 4.29)	0.0899
B_h-RF_	87	34	57 (46– 70)		2.75 (1.42–5.32)	0.0027**	2.84 (1.23 - 6.57)	0.0146*
*CAE*				0.0028**				
B_CAE_ (ref.)	87	18	81 (72– 90)		*reference*	–	*reference*	–
A_CAE_	138	52	56 (48– 67)		2.23 (1.3 - 3.82)	0.0035**	1.50 (0.79 - 2.85)	0.2112
*HSCAE*				<0.0001***				
B_HSCAE_ (ref.)	89	15	84 (76– 93)		*reference*	–	*reference*	–
A_HSCAE_	136	55	54 (45– 64)		3.29 (1.85–5.84)	<0.0001***	2.73 (1.37– 5.42)	0.0043**

Covariables used in the multivariable Cox regression were: histological grade, histological type (categorized as in the SARCULATOR nomogram), age, size, use of adjuvant or neoadjuvant anthracyclines-based chemotherapy, use of adjuvant radiotherapy, and surgical margins.

*CI* confidence interval, *CAE* convolutional autoencoder neural network, *h-RF* handcrafted radiomics features, *HR* hazard ratio, *HSCAE* half supervised convolutional autoencoder neural network, *MFS* metastatic relapse-free survival, *No.* number, *ref.* reference level.

*P < 0.05, **P < 0.005, ***P < 0.001. Significant results are in bold.

Hence, regarding the h-RF clustering, hazard ratios (HRs) were 1.97 (95% confidence interval [CI]: 0.90-4.29, *P* = 0.0899) for A_h-RF_ group and 2.84 (95% CI: 1.23-6.57, *P* = 0.0146) for B_h-RF_ group, compared to the C_h-RF_ group.

Regarding d-RF clustering, multivariable analyses provided HR = 1.50 (95% CI: 0.79–2.85, *P* = 0.2112) for A_CAE_ group compared to B_CAE_ group, and HR = 2.73 (95% CI: 1.37–5.42, *P* = 0.0043) for A_HSCAE_ group compared to B_HSCAE_ group.

**Fig. 2:**
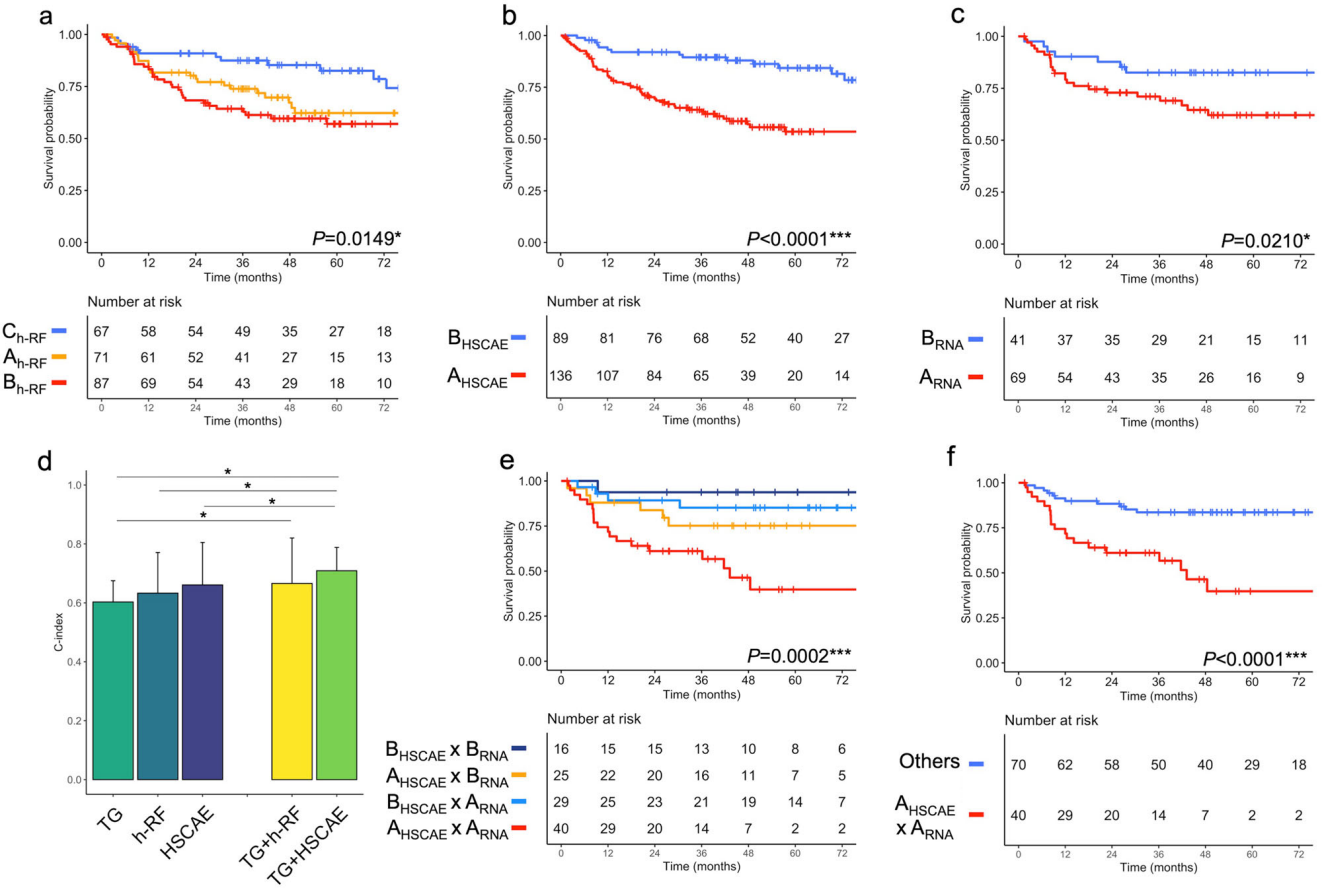
Summary of the survival analyses for metastatic relapse free survival (MFS) in the subcohort of patients with both radiomics and transcriptomics data (*n* = 110). Kaplan–Meier curves (with their risk tables) for (**a**) unsupervised clustering based on handcrafted radiomics features (h-RF groups), for (**b**) unsupervised clustering based on the half-supervised convolutional autoencoder neural network deep radiomics features (d-RF HSCAE groups) and for (**c**) the unsupervised transcriptomics groups (TG). The panel (**d**) shows the 5-fold cross-validated concordance-indices (c-index, with 95% confidence interval) for Cox regression models based on the h-RF, HSCAE, and transcriptomics groups alone and combined. **e**, **f** Creation of a binary hybrid variable accounting for the d-RF HSCAE and transcriptomics groups (exhaustive and binarized). *P*-values correspond to the Log-rank tests. *P < 0.05, ***P < 0.001.

Stepwise Cox regression selected the HSCAE clustering for further analyses in its last step (minimal Akaike information criterion [AIC] = 645.8). Kaplan–Meier curves for h-RF and HSCAE clustering are presented in Fig. 2a, b.

### Complementarity of radiomics and transcriptomics

The complementarity of h-RF clustering and the relevant d-RF clustering (HSCAE) with transcriptomics clustering was assessed in 110 patients having both the radiomics and transcriptomics data.

Transcriptomics clustering was associated with MFS in univariable analysis (HR for A_RNA_ of 2.60, 95% CI: 1.12–6.02, *P* = 0.0261) (Table 4, Fig. 2c). B_h-RF_ and A_HSCAE_ groups remained associated with lower MFS in this subcohort (univariable HR = 3.55, 95% CI: 1.29–9.72, *P* = 0.0140, and HR = 3.90, 95% CI: 1.60–9.52, *P* = 0.0028, respectively).

**Table 4:** Survival analysis and predictive performances in the subcohort of *n* = 110 patients with both radiomics and transcriptomics data

Characteristics	No. at risk	No. of events	5-year MFS probability	Log Rank *P*-value	HR (95%CI)	*P*-value	C-index (5 fold CV)
*Univariable model*							
Transcriptomics groups				0.0210*			0.603 (0.574–0.675)
B_RNA_="vertical-align: inherit;"> (ref.)	41	7	83 (72–95)		*reference*	–	
A_RNA_	69	25	62 (51–76)		2.60 (1.12– 6.02)	0.0261*	
*h-RF groups*				0.0263*			0.633 (0.599–0.771)
C_h-RF_ (ref.)	34	5	85 (73–98)		*reference*	–	
A_h-RF_	38	11	71 (57–89)		1.94 (0.67– 5.58)	0.2210	
B_h-RF_	38	16	56 (41–76)		3.55 (1.29– 9.72)	0.0140*	
*d-RF groups (HSCAE)*				0.0013**			0.661 (0.615–0.805)
B_HSCAE_ (ref.)	45	6	88 (79–99)		*reference*	–	
A_HSCAE_	65	26	56 (43–71)		3.90 (1.60– 9.52)	0.0028**	
*Combined model (h-RF)*							0.666 (0.643–0.820)
Transcriptomics groups (B_RNA_) (ref.)	–	–	–	–	*reference*	**–**	
Transcriptomics groups (A_RNA_)	–	–	–	–	3.08 (1.32– 7.17)	0.0092*	
Radiomics groups (C_h-RF_) (ref.)	–	–	–	–	*reference*	–	
Radiomics groups (A_h-RF_)	–	–	–	–	2.02 (0.70– 5.83)	0.1921	
Radiomics groups (B_h-RF_)	–	–	–	–	4.23 (1.54– 11.65)	0.0052*	
*Combined model (d-RF)*							0.709 (0.651–0.788)
Transcriptomics groups (B_RNA_) (ref.)	–	–	–	–	*reference*	**–**	
Transcriptomics groups (A_RNA_)	–	–	–	–	2.93 (1.25– 6.83)	0.0129*	
HSCAE (B_HSCAE_) (ref.)	–	–	–	–	*reference*	–	
HSCAE (A_HSCAE_)	–	–	––		4.28 (1.74 - 10.50)	0.0015**	
*Simplified combined model (d-RF)*^**§**^				<0.0001***			
Others (ref.)	70	12	84 (75–93)		*reference*	**–**	0.689 (0.620-0.814)
A _HSCAE_ A _R NA_	40	20	40 (24–66)		4.29 (2.07–8.90)	<0.0001****	

*C-index* concordance index, *CI* confidence interval, *CAE* convolutional autoencoder neural network, *CV* cross validation, *d-RFs* deep radiomics features, *h-RFs* handcrafted radiomics features, *HR* hazard ratio, *HSCAE* half-supervised convolutional autoencoder neural network, *MFS* metastatic relapse-free survival, *no.* number, *ref.* reference level.

^§^In the simplified combined model, the A_HSCAE_ and A_RNA_ groups, with the lowest MFS, were pooled against all the other groups of patients to constitute a meaningful binary variable usable for further gene-expression analysis.

*P < 0.05, **P < 0.005, ***P < 0.001. Significant results are in bold.

In a Cox regression model including the h-RF clustering and the transcriptomics clustering, the A_RNA_ and B_h-RF_ groups were still associated with lower MFS (HR = 3/08, 95% CI: 1.32-7.17, *P* = 0.0092, and HR = 4.23, 95% CI: 1.54-11.65, *P* = 0.0052, respectively).

Similarly, in a Cox regression model including the HSCAE clustering and the transcriptomics clustering, the A_RNA_ and A_HSCAE_ groups remained associated with lower MFS (HR = 2.93, 95% CI: 1.25-6.83, *P* = 0.0129, and HR = 4.28, 95% CI: 1.74-10.50, *P* = 0.0015, respectively).

In order to identify the most relevant combination of radiomics and transcriptomics clustering, we then investigated and compared the prognostic value of univariable and combined models using a 5-fold cross-validation scheme. Significant increment in Harrell concordance index (c-index) with radiomics-based clustering combined with transcriptomics clustering was observed (Fig. 2d). Thus, the c-index for the Transcriptomics groups alone was 0.603 (95% CI: 0.574–0.675), versus 0.633 (95% CI: 0.599–0.771) for h-RF group alone and 0.666 (95% CI: 0.643–0.820) for the Transcriptomics × h-RF combined model (*P* = 0.0380 against Transcriptomics group alone, and *P* = 0.0469 against h-RF group alone). Regarding deep radiomics, the c-index was 0.709 (95% CI: 0.651–0.788) for the Transcriptomics × HSCAE combined model, which was significantly higher than for the HSCAE model alone (c-index = 0.661, 95% CI: 0.615–0.805, *P* = 0.0220) and for Transcriptomics group alone (*P* = 0.0110).

Consequently, we selected the HSCAE and transcriptomics combined model. Kaplan–Meier analysis reveals that the A_RNA_ × A_HSCAE_ combination group has a worse MFS prognosis with respect to all the other combination groups (hereafter called the ‘Other’ group, made of the BRNA × A_HSCAE_, A_RNA_ × B_HSCAE_ and BRNA × B_HSCAE_ combinations) (Fig. 2e, f). The HR for the ARNA × A_HSCAE_ group against other Other groups was 4.29 (95% CI: 2.07–8.90, *P* < 0.0001, Table 4). The ARNA × A_HSCAE_ group remains significantly associated with MFS in the most frequent histotype, namely UPS (*n* = 29, with HR = 8.77, 95% CI: 1.04–73.6, *P* = 0.0456). We proceeded then with the differential gene expression analysis between the A_RNA_ × A_HSCAE_ group and the Other group.

Lastly, we investigated whether the prognostic performance of the clinical–histological SARCULATOR nomogram would be increased with deep radiomics and transcriptomics. The average c-indices in 5-fold cross-validation increased from 0.698 (95% CI: 0.608–0.787) for the SARCULATOR groups alone to 0.728 (95% CI: 0.650–0.807) for a model combining the SARCULATOR, HSCAE, and transcriptomics groups, despite the difference is not significant (*P* = 0.1458). In this last Cox model, two characteristics remained independently associated with MFS: the high Pr-OS SARCULATOR group (HR = 6/04, 95% CI: 2.48–14.73, *P* < 0.0001) and the A_HSCAE_ group (HR = 2.69, 95% CI: 1/01–7/14, *P* = 0.0477). The Supplementary Table ST3 shows the corresponding survival table for this sub-analysis.

### Gene-expression analysis

**Fig. 3:**
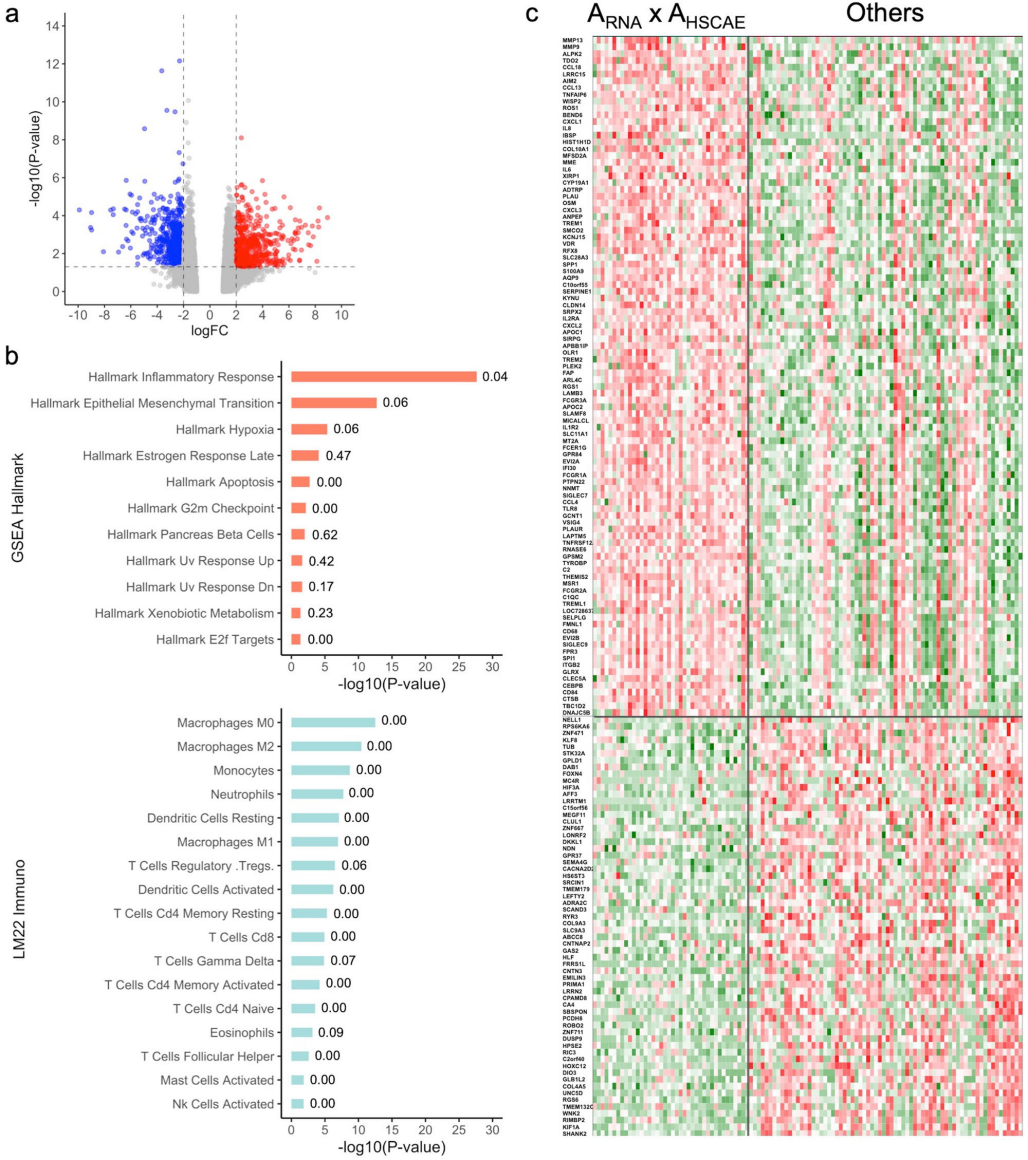
Summary of the differential gene expression (DGE) and pathway analyses between the A_RNA_ × A_HSCAE_ group and the ‘Others’ groups. Volcano plot of the DGE analysis (**a**). Significantly enriched genes and pathways in the A_RNA_ × A_HSCAE_ group are in red, whereas those significantly downregulated are in blue. **b** Summary report of the most significantly enriched gene sets belonging to the HALLMARK of Cancer and the LM22 human immune cells collections; values on the right side of the bars correspond to the proportion of genes activated in the ‘Other’ groups. **c** Heatmap of the 162 activated and inhibited genes from the PAMr cross-validated discriminant signature of the A_RNA_ × A_HSCAE_ group versus the ‘Others’ groups. All *P*-values on the plots were adjusted according to the Benjamini–Hochberg procedure. FC fold change.

The Volcano plot analysis of differential gene expression (DGE) between A_RNA_ × A_HSCAE_ and Other groups identified 1230 differentially expressed genes (Fig. 3a). The full list of genes and their associated annotations are reported in Supplementary Table ST4. GeneSet enrichment analysis on the differentially expressed genes revealed 292 significantly enriched pathways (Supplementary Table ST5 reports the description of the gene set, the adjusted *p*-value, the enrichment status, and the activation or inhibition status of the other group). Figure 3b shows the list of the most significantly enriched pathways. The analysis of the genes participating in those genesets showed that the A_RNA_ × A_HSCAE_ group of worse prognosis activates the inflammatory response, epithelial-mesenchymal transition, hypoxia, apoptosis inhibition, G2M checkpoint, UV response, E2F targets, and xenobiotic metabolism gene sets. We performed an extensive review of the role of the genes belonging to those pathways whose results are reported in Supplementary Table ST6. For each geneset, we reviewed the description of the participating genes, their role played in a specific tumor type and the reference to the scientific study showing evidence of this role. We identified important genes having an oncogenic role in a wide variety of tumors where the role of the gene seems to be coherent with the one played in the A_RNA_ × A_HSCAE_ group of worse prognosis. A discriminant signature between the A_RNA_ × A_HSCAE_ and other groups, performed via PAMr, a machine learning method, identified a subset of 162 discriminant genes (Fig. 3c). The detailed annotation of those genes (reported in Supplementary Table ST7) highlighted genes belonging to the epithelial–mesenchymal transition, hypoxia, and apoptosis inhibition genesets in the A_RNA_ × A_HSCAE_ group.

**Fig. 4:**
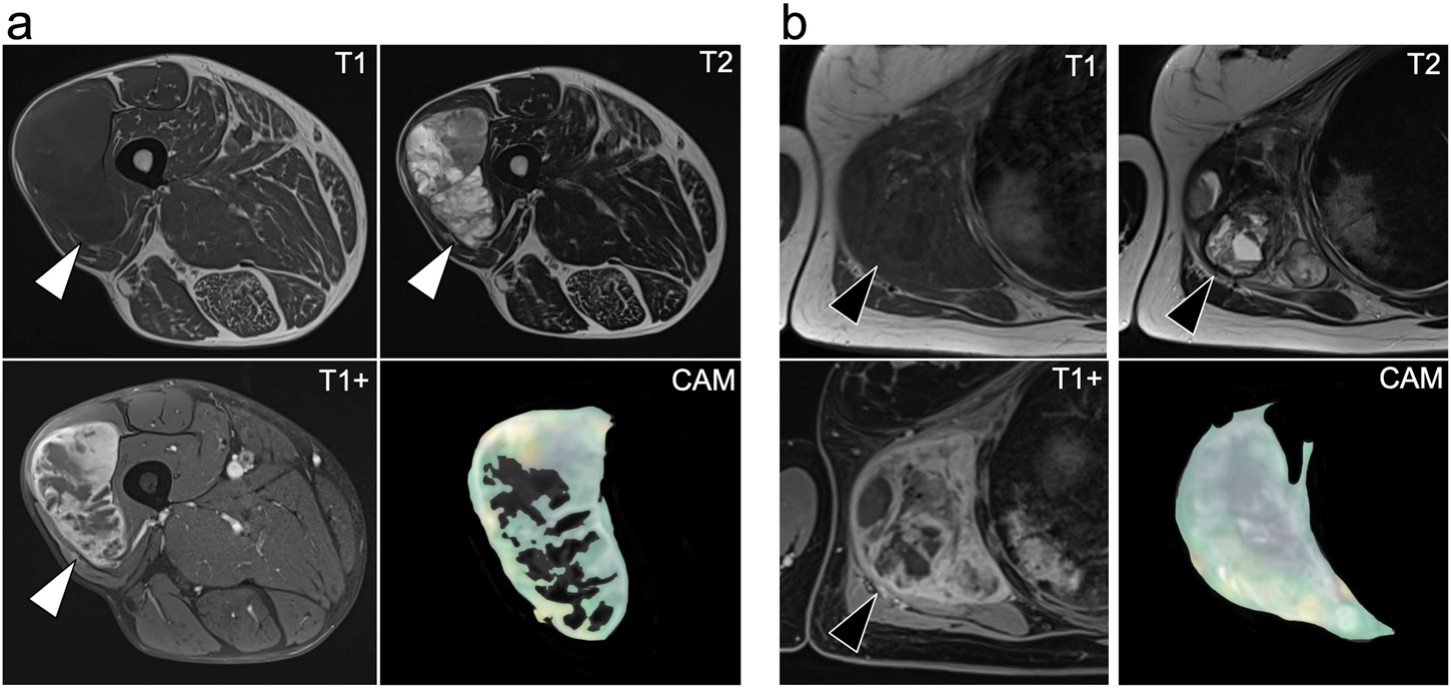
Examples. **a** A 67 years old man was diagnosed with a deep-seated, 89 mm long, FNCLCC grade III leiomyosarcoma of the thigh (white arrowheads). The tumor was classified in the A_HSCAE_ group as well as in the A_RNA_ group (i.e., hybrid group associated with worse survivals). The patient showed a lung metastatic relapse 8 months after diagnosis and died of disease 4 months later. **b** A 51 years old woman was diagnosed with a deep-seated, 115 mm long, FNCLCC grade II (on biopsy) dedifferentiated liposarcoma of the shoulder (black arrowheads). The tumor was classified in the B_HSCAE_ group as well as in the B_RNA_ group (i.e., groups associated with higher survivals). The patient has shown no relapse at the last follow-up, i.e., 5 years after diagnosis, and is still alive. Thus, although being rather similar for human eyes on conventional MRI, combining deep radiomics and transcriptomics data enabled us to distinguish these two patients with opposite outcomes. CAM class activation mapping (Supplementary Method M1) highlighting the important areas for predictions made by the half-supervised autoencoder neural network (HSCAE), T1: T1 weighted imaging, T2: T2 weighted imaging, T1+ contrast-enhanced fat-suppressed T1-weighted imaging.

MRI examples of patients with opposite radiomic-transcriptomics grouping and outcomes are presented in Fig. 4.

## Discussion

In this study, we devised methods to classify soft tissue sarcomas (STS) imaging, independent of their initial presentation on conventional MRI, employing both handcrafted radiomics (h-RFs) and deep radiomics (d-RFs, using CAE and HSCAE models). Cluster analysis identified classes that were consistently correlated with the clinical and histological SARCULATOR nomogram and prognostic semantic radiological phenotypes while displaying a notable disconnection from transcriptomic clusters^19^. Our findings suggest that combining pre-treatment radiomics and transcriptomic data through a combined radiomic-transcriptomic signature could enhance the predictive performance of transcriptomics in STS patients.

To our knowledge, no study has concurrently analyzed radiomics and transcriptomic datasets in a large cohort of STS patients. Radiomics data, obtained through either handcrafted or deep radiomics approaches, demonstrated strong associations with semantic radiological features linked to aggressive tumor characteristics, higher grade, and poorer survival outcomes^3,4^. Moreover, the h-RF grouping was significantly associated with the histological types, which illustrates the potentiality of imaging to pre-type STS from radiological images directly. The d-RF groups additionally correlated with FNCLCC grade and SARCULATOR groups, i.e., with histological and clinical variables strongly associated with metastasis and death risk. Collectively, these associations between radiomics groups, identified without prior assumptions, and clinical, histological, and radiological features having prognostic significance contribute to validating the relevance of radiomics data.

Notably, although the fully unsupervised h-RFs, the CAE clustering, and the HSCAE clustering exhibited c-indices lower than some previously published supervised models, they remained significantly better than random in both univariable and multivariable analyses accounting for cofounding covariables underscoring their intrinsic prognostic value for STS patients^7,14,18,20–22^.

Transcriptomics groups, while exhibiting weaker but significant associations with patient survivals, showed a substantial increase in prognostic performance (c-index = 0.709) when combined with HSCAE groups. The Kaplan–Meier curves for the combined deep-radiomics-transcriptomics variable demonstrated a clear gradient in survival probability from less to more aggressive groups. This emphasizes the complementary nature of radiomics and transcriptomics, suggesting that adding radiomics data to prognostic gene-expression signatures could enhance their predictive value. We believe this complementarity could be explained by the difference and complementarity in scale and nature of radiomics and transcriptomics data. Indeed, radiomics are macroscopic data assessed over a digital image of the entire tumor volume, whereas transcriptomics data are extracted from millimetric tumor samples. Even though our findings do not provide immediate clinical application, we believe they should encourage the investigations of multi-omics prognostic signatures for STS patients through multi-center collaborations. Moreover, our findings also suggest that radiomics-transcriptomics data could enhance pre-existing clinical-histological nomograms such as the SARCULATOR.

Lastly, our attempt to elucidate the gene-expression levels of the A_RNA_ × A_HSCAE_ group (indicating worse outcomes) involved analyzing 1230 differentially expressed genes (including 707 overexpressed genes) and 262 pathways in 110 patients. These findings implicated the tumor micro-environment and tumorigenesis through the dysregulation of adaptive immune reactions, cell growth, inflammation, cell differentiation, apoptosis, and hypoxia^16^^,22^. Interestingly, although the transcriptomics groups were significantly associated with the histological types, the A_RNA_ × A_HSCAE_ group remains associated with significantly lower MFS in the most frequent histotype, i.e., UPS. It must be emphasized that gene-expression signatures dedicated to STS exist, notably the complexity index in sarcoma (CINSARC) signature^16,23^, which would enhance the prognostic performances of the SARCULATOR nomogram^24^. The aim of this study was not to challenge CINSARC (established on 310 samples versus 110 in our study). However, we found 17 common underlying pathways between CINSARC and the pathways identified in our transcriptomics analysis (Supplementary Table ST8), as well as strong associations (*P* = 0.0069, Chi-Square test) between CINSARC and the transcriptomics groups in a subset of 54/110 (49.1%) patients with available data (unshown data). This highlights the consistency of molecular features involved in the aggressiveness of STS.

Despite these valuable insights, our study has limitations, including its retrospective nature, heterogeneous imaging protocols necessitating MRI processing, and the availability of transcriptomic data for only half of the study population. Additionally, deep radiomics analyses were restricted to a central slice of CE-T1-WI due to current limitations in freely available deep learning algorithms for multiple co-registered 3D volumes. Lastly, although large compared to prior radiomics studies in STS, the size of the study population was too small to enable the development of a reliable and more powerful supervised deep-learning model to predict patient MFS. For instance, the DeepSurv model required datasets with more than 1500 observations for its training^25^. Similarly, even though the MSEs of the reconstructions by the CAE and HSCAE were high in the Training and Testing cohorts, the small size of the Testing cohort (*n* = 25 patients) suggests the need to validate the approach in larger independent cohorts. Addressing these limitations could further enhance the performance of deep-learning models.

In conclusion, our study provides a comprehensive analysis of MRI radiomics profiles for STS, revealing relationships with patient outcomes, semantic radiological features, and the SARCULATOR nomogram. Moreover, it highlights the synergistic potential of radiomics and transcriptomic data to refine the prognostication of STS patients, opening avenues for the development of a radiomic-transcriptomic prognostic signature for sarcoma patients.

## Methods

### Study design

This single-center study was approved by the institutional review board of Bergonié (Sarcoma Reference Center of Nouvelle-Aquitaine, Bordeaux, France). Written informed consent was waived by its retrospective nature. The study was achieved in agreement with good clinical practice and applicable laws. All research procedures and protocols adhered to the principles set forth in the Declaration of Helsinki.

Patients were identified by the prospectively held surgical database from our sarcoma reference center. We included all consecutive patients between May 2008 and May 2020 as they presented with a newly diagnosed locally advanced STS, with histopathological confirmation according to a senior pathologist with expertise in STS from our institution (F.L.L., J.M.C., and R.P.), with available pre-treatment MRI including a gadolinium-chelates injection, and treated in a curative intent including surgery.

We excluded patients with atypical lipomatous tumors, metastases at initial staging (i.e., on chest CT scan), and patients whose pre-treatment MRI did not include at least one T1-weighted imaging (WI), one T2-WI, and one fat-suppressed (FS) CE T1-WI (CE-T1-WI).

**Fig. 5:**
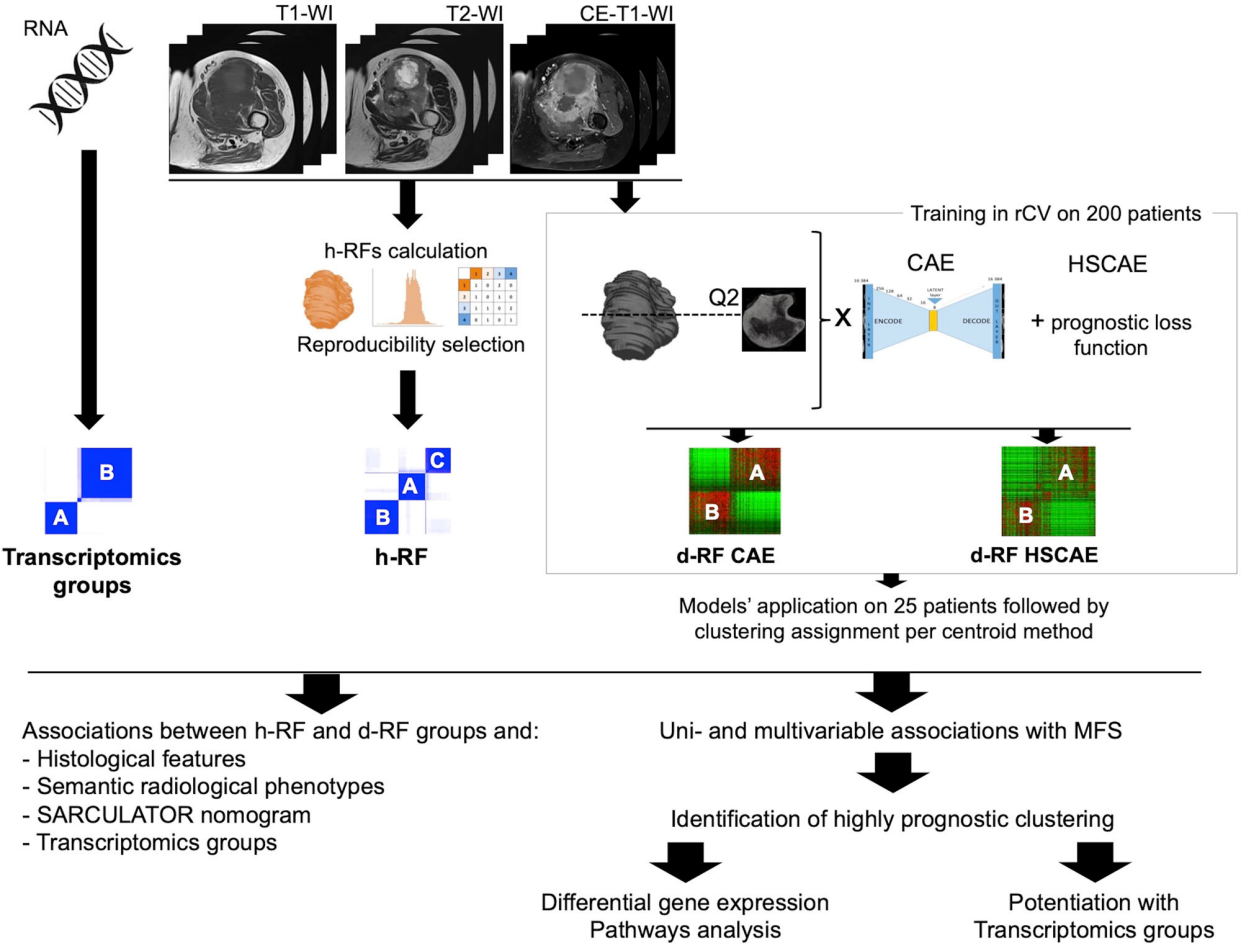
Analysis workflow. CE contrast enhanced, CAE convolutional autoencoder neural network, d-RF deep radiomics features, h-RF handcraft radiomics features, HSCAE half-supervised convolutional autoencoder neural network, MFS metastatic relapse-free survival, rCV repeated cross-validation, RNA ribonucleic acid, WI weighted imaging.

Figure 1 shows the flow chart. Figure 5 shows the overall study workflow.

### Histologic and clinical data collection

Data collection from medical records comprised the patient’s age at diagnosis, sex, World Health Organization Performance status (WHO-PS), the tumor depth, location, longest diameter (LD), pre-treatment histological type, and FNCLCC grade (either on biopsy or on entire specimen)^26^, and the initial therapeutic management, namely radiotherapy or anthracycline-based chemotherapy (categorized as none, neoadjuvant or adjuvant) always combined with curative surgery, and the surgical margins (categorized as R0 versus R1–R2).

Moreover, the 10-year predicted probability of OS (Pr-OS) according to the SARCULATOR nomogram for extremity and trunk wall STS was calculated for each patient using the free application^19^. Patients were then divided into three categories of 10-year Pr-OS: low (≤51%), intermediate (>51% and ≤66%), and high (>66%), as previously described in prior studies from the same authors^27^^, 28^. The corresponding new categorical variable was named SARCULATOR.

Follow-ups consisted of clinical examinations and chest radiographs every 3 months for 2 years, then every 6 months for 5 years, and then annually, with complementary local MRI and chest CT-scan in case of doubtful findings. The main outcome was MFS. MFS, local relapse-free survival (LFS), and overall survival (OS) corresponded to the time (in months) elapsed from surgery to metastatic relapse, local relapse, and death related to disease (or last follow-up), respectively. Patients without events during the study period were censored. All relapses were histologically proven.

### MRI acquisition

The pre-treatment MRI examinations were acquired on different 1.5-Tesla MR-systems with adjustments of the coils, field-of-views, and matrices depending on tumor size, location, and depth. The protocols systematically included at least one T1-WI prior to contrast injection, one T2-WI, and one FS CE-T1-WI. Various methods were accepted for FS, i.e., fat saturation, Dixon, subtraction, fluid-sensitive, and short tau inversion-recovery sequences^29^. The ranges of repetition time and echo time were 500–700 and 10–15 ms for T1-WI and 2400–4500 and 70–130 ms for T2-WI, respectively. The ranges of in-plane resolution and thickness were 0.75 × 0.75–1.4 × 1.4 mm^2^ and 1–7 mm, respectively.

### Conventional radiological analysis

The conventional (or semantic) radiological analysis was performed in consensus by two senior radiologists with expertise in STS (A.C. and M.K.) on a picture archiving and communication system (Enterprise Imaging, Agfa Healthcare, Mortsel, Belgium). They reproduced the same analysis as in the prognostic study by Crombé et al.^4^ and reported if at least 2 out of the 3 following semantic radiological features were present: (i) heterogeneous signal intensity (SI) on T2-WI ≥ 50% (i.e., when ≥50% of the tumor volume showed areas with high, intermediate and low SI), (ii) presence of an area compatible with necrosis (i.e., defined as high fluid-like SI on T2-WI without enhancement at T1-WI after gadolinium chelate injection), and (iii) presence of peritumoral enhancement (i.e., defined as contrast enhancement beyond the apparent tumor borders without mass effects), as the resulting semantic radiophenotype (named ‘high risk’) was a significant predictor of lower MFS and OS in this study. Conversely, if one or less of these three radiological features was seen, the tumor was categorized as ‘low risk’.

### Radiomics groups

#### Handcrafted radiomics pipeline

Since the sequences were obtained on different MR-systems and given the lack of standardized units for conventional MRI, a 3-steps post-processing pipeline was achieved on the T1-WI, T2-WI, and CE-T1-WI sequences in order to homogenize the imaging dataset using the ITK library (https://github.com/InsightSoftwareConsortium/ITK). First, a bilinear interpolation was applied to resample the voxel size to a common resolution of 1 × 1 × 4 mm^3^. Second, non-uniform SIs due to magnetic field heterogeneity were corrected using N4-ITK bias field correction^30^. Third, SIs were homogenized with simple ITK histogram-matching and ranged between −10,000 and +10000^31^. Afterward, the MRIs were uploaded to the LIFEx freeware (version 4.70, Saclay, France), compliant with the guidelines from the International Biomarker Standardization Initiative^32,33^. One senior radiologist manually segmented the entire tumor volumes of interest (VOIs), slice-by-slice, on the CE-T1-WI and then propagated this VOI on the T1-WI and T2-WI with adjustments of the segmentation boundaries if needed. Afterward, the SIs were discretized into 256 gray-levels and 59 texture h-RFs were extracted per sequence, namely 13 first-order texture h-RFs, 46 second-order texture h-RFs (21 from the gray-level co-occurrence matrix [GLCM] using 1, 2 and 4 pixel distance to neighbors; 11 from the gray-level run length matrix [GLRLM], 3 from the neighborhood gray-level difference matrix [NGLDM], and 11 from the gray-level zone length matrix [GLZLM]) in addition to 4 shape features from CE-T1-WI. The precise definitions and formulas for all RFs can be found at: https://www.lifexsoft.org/index.php/resources/19-texture/radiomic-features.

The same procedure (from segmentation to RF extraction) was reproduced on 30 randomly selected patients in order to estimate the reproducibility of RFs according to intra-class correlation coefficient (ICC). We only selected the RFs with ICC ≥ 0.90 for the remaining analyses, namely 46 texture h-RFs from T1-WI, 52 texture h-RFs from T2-WI, 52 texture h-RFs from CE-T1-WI and 2 shape h-RFs (i.e., *n* = 152 h-RFs, listed in Supplementary Table T9). The consensus clustering algorithm was applied to the robust h-RFs from the entire cohort. After center-scaling those h-RFs, each clustering was resampled 10,000 times by leave-one-out of 40% of the samples, based on hierarchical clustering using the Pearson distance and the average link^34^.

#### Deep radiomics pipeline

For each patient, the mask resulting from the tumor segmentation was propagated on the initial fat-suppressed CE-T1-WI to remove the background surrounding the tumor with manual adjustments made if necessary. Subsequently, the slice at the tumor’s midpoint was selected for further analysis. Two types of neural networks were built: convolutional auto-encoder neural network (CAE) and half-supervised convolutional auto-encoder neural network (HSCAE), which included a prognostic loss function. Details on data preprocessing, augmentation, model architecture, loss function, optimization, performance evaluation, and d-RF extraction for each network are provided in Supplementary Method M1^35–38^. Overall, the output of a convolutional autoencoder neural network is an image that is the best ‘replicate’ of the input image. To do so, the network articulates in two sequential sub-networks. The first is a ‘decoder network’, similar to a classical convolutional neural network used for supervised classification of images, where the input image is progressively reduced to a vector of ‘latent features’. The second part is an ‘encoder network’ which takes the ‘latent features’ and progressively reconstructs the input image by deconvolution. The fidelity of the reconstruction was optimized using the mean square error (MSE) between the input and output data. We trained the CAE and HSCAE models using a leave-10%-out cross-validation technique on a randomly selected subset of 200 patients (Training cohort), repeating the process 100 times. The resulting vector of ‘latent features’ (i.e., 1024 d-RFs per image) was subjected to unsupervised hierarchical clustering (with the Pearson distance and average link, after center-scaling the d-RFs) to assign each patient to a cluster (CAE and HSCAE grouping). Additional methodological details notably about artefactual morphological augmentation techniques, are given in Supplementary Method M1. Regarding the 25 remaining patients from the independent test set of 25 samples (Testing cohort), we verified the quality of the reconstructions provided by CAE and HSCAE according to MSE. Afterward, their CAE and HSCAE clusters were secondarily identified using the centroid method, which consists of calculating the Pearson distance between each new observation and the centroid of each cluster and to attribute the cluster with the shortest distance.

### Gene expression analysis

We included in the analysis all patients with frozen material or paraffin-embedded tissue of good quality before treatment and contemporary to the baseline MRI used for radiomics. After whole-RNA sequencing, the produced RNA sequences were quality-controlled and aligned to the transcriptome. Gene Expression was then estimated by the counts of high-quality sequences aligned per Gene. ComBat harmonization method was applied to correct batch effect^39^. Finally, gene expression counts were normalized using the Voom method (Supplementary Method M2)^40^. Unsupervised transcriptomics grouping was similarly built on RNA-sequencing data with hierarchical consensus clustering.

### Statistical analyses

Statistical analyses were also performed with R (v4.1.0). All tests were two-tailed. A *P*-value < 0.05 was deemed significant.

#### Understanding the ‘omics’ groups

Associations between the h-RFs group, d-RFs CAE and HSCAE groups, transcriptomics groups, histologic types, histologic grade, SARCULATOR groups, and semantic radiophenotypes (i.e., categorical variables) were tested with Chi-square tests. Associations with the SARCULATOR Pr-OS and tumor size were investigated using unpaired *t*-test of the Mann–Whitney test depending on the Shapiro–Wilk normality test.

#### Prognostic value of the ‘omics’ group alone and in combinations

The Kaplan–Meier curves for MFS, depending on the radiomics and transcriptomics groups, were drawn, and the differences in survivals were tested with the log-rank test. Univariable and multivariable Cox regressions were performed to estimate the hazard ratio (HR) with a 95% confidence interval (CI) of each group. Multivariable models comprised the following covariables involved in the SARCULATOR nomograms and accounting for patient management: age (continuous), histologic type (according to the SARCULATOR categorization, with myxoid/round cells liposarcoma as reference), histologic grade (I and II [reference] versus III), tumor size (continuous), chemotherapy (no [reference] versus yes) radiotherapy (no versus yes [reference]), and surgical margins (R0 [reference] versus R1 and R2). To identify the most relevant deep-RF grouping among the three we developed, a stepwise backward Cox regression (minimizing the Akaike information criterion [AIC]) including all d-RF clusters and the covariables was built. Lastly, to evaluate the complementarity of radiomics groups and transcriptomics groups to predict patient outcome, the Harrell concordance indices (c-indices) of the best d-RF grouping alone, the h-RF grouping alone, the transcriptomics grouping alone, and their combination (with and without interaction term) were evaluated in 5-fold cross-validation and compared using a bootstrapped distribution of their difference over 1000 random replicates of the study population. The same approach was applied to evaluate the potential synergy between the SARCULATOR, Transcriptomics, and d-RF groups. Patients with any missing value among the input variables of the multivariable Cox regressions were removed from the analyses.

#### Gene-expression profiling of the prognostic radiomics groups

Differential gene expression (DGE) and geneset enrichment analyses were performed between the radiomics-transcriptomics grouping with the highest association with patient MFS. Exploratory DGE was performed by *t*-test calculation per gene. To discriminate significant up/down-regulated genes, the fold change was set to 2. The *P*-value cut-off was adjusted with the Benjamini–Hochberg procedure. We then assessed geneset enrichment in biological pathways based on Broad Institute’s Molecular Signature database and the CIBERSORT LM22 immuno-genesets (Supplementary Method M3)^41,42^. Lastly, the prediction analysis for microarrays (PAM) method was applied to provide class prediction from gene expression profiling based on an enhancement of the simple nearest prototype (centroid) classifier (Supplementary Method M3)^43–45^.

### Reporting summary

Further information on research design is available in the Nature Research Reporting Summary linked to this article.

### Supplementary information

Supplementary Materials

Reporting Summary

## Data Availability

The raw and processed data generated in this study have been deposited in NCBI’s Gene Expression Omnibus (GEO) and are accessible through GEO Series accession number GSE262937 for RNA-seq experiment. The differential gene expression analyses and pathways analyses are available in the supplementary materials. The radiomics datasets and raw MRIs used and/or analyzed during the current study are available from the corresponding author on reasonable request. Any additional results can be obtained from the corresponding author.

## Code availability

The underlying code for this study is not publicly available but may be made available to qualified researchers on reasonable request from the corresponding author. The R studio and Conda environments and the packages with their versions used for the analyses are detailed in Supplementary Table ST10.
