# Supplementary material for: Integration of pre-treatment computational radiomics, deep radiomics, and transcriptomics enhances soft-tissue sarcoma patient prognosis
Source: NPJ Precis Oncol. 2024 Jun 7;8:129. doi: 10.1038/s41698-024-00616-8 (PMC11161510; doi:10.1038/s41698-024-00616-8)
Supplement: Supplementary file 1 — Supplementary Materials [file 41698_2024_616_MOESM1_ESM.pdf]

SUPPLEMENTARY DATA

Supplementary Table ST1. Mean square error (MSE) for the reconstruction (or output) of the input image(s) provided to the autoencoder neural networks (CAE and HSCAE) in the Training and Testing cohorts.

Model	MSE	
	**Training cohort**	**Testing cohort**
CAE	0.0026	0.0200
HSCAE	<10-7	0.0022

Supplementary Table ST2. Univariable survival analysis for the covariables in the whole cohort.

Characteristics	No. at risk	No. of events	5-year MFS probability	Log-rank *P*-value	Univariable HR (95% CI)	*P*-value
**Age**				0.6782		
< median (62 years)	112	34	66 (56 - 77)		-	-
≥ median	113	36	67 (58 - 77)		1.1 (0.69 - 1.77)	0.6779
**Histologic type**				0.1378		
M/RCLPS	22	6	63 (40 - 97)		-	-
Pleo./DD LPS	27	7	75 (59 - 95)		0.94 (0.31 - 2.79)	0.9050
Leiomyosarcoma	24	11	48 (28 - 81)		2.10 (0.78 - 5.69)	0.1440
Myxofibrosarcoma	13	0	100 (100 - 100)		0 (0 - Inf)	0.9941
Synovial sarcoma	16	6	62 (42 - 91)		1.57 (0.50 - 4.86)	0.6302
UPS	71	23	66 (55 - 79)		1.25 (0.51 - 3.07)	0.4369
other	52	17	64 (51 - 81)		1.36 (0.54 - 3.46)	0.5170
**FNCLCC grade**				**0.0014****		
Grade I and II	106	24	76 (66 - 86)		-	-
Grade III	116	46	57 (49 - 68)		2.2 (1.34 - 3.6)	**0.0018****
**Size**				**0.0336***		
< median (87 mm)	111	28	75 (66 - 84)		-	-
≥ median	114	42	58 (48 - 69)		1.004 (1 - 1.008)	0.0520
**Radiotherapy**				0.5738		
No	31	7	75 (58 - 96)		-	-
Yes	194	63	65 (58 - 73)		1.25 (0.57 - 2.73)	0.5742
**Chemotherapy**				**0.0243***		
No	101	24	76 (67 - 85)		-	-
Yes	124	46	59 (50 - 70)		1.75 (1.07 - 2.88)	**0.0263***
**Surgical margins**				**0.0021****		
R0	144	34	74 (66 - 83)		-	-
R1-R2	77	34	54 (43 - 68)		2.08 (1.29 - 3.34)	**0.0026****

NOTE.- *: *P*<0.05, **: *P*<0.005, ***: *P*<0.001. Significant results are in bold.
Other abbreviations: CI: confidence interval, DD: dedifferentiated, FNCLCC: French federation of cancer centers, HR: hazard ratio, LPS: liposarcoma, M/RC: myxoid/round cells, No.: number, UPS: undifferentiated pleomorphic sarcoma

Supplementary Table ST3. Survival analysis in the subcohort of n= 110 patients with both radiomics and transcriptomics data: assessment of the added value of the HSCAE transcriptomics groups to the SARCULATOR groups

Characteristics	No. At risk	No. of events	5-year MFS probability	Log-rank P-value	HR (95%CI)	P-value	c-index (95%CI)
**Univariable Model**							
**SARCULATOR groups**				**<0.0001***			
High Pr-OS (ref.)	53	7	88 (80 - 98)		*reference*	-	0.698 (0.608-0.787)
Interm. Pr-OS	26	9	63 (47 - 86)		2.98 (1.11-8.01)	**0.0396***	
Low Pr-OS	29	16	40 (24 - 68)		6.04 (2.48-14.73)	**<0.0001***	
**Combined Model**							
**SARCULATOR groups**							
High Pr-OS (ref.)	-	-	-	-	*reference*	-	0.728 (0.650-0.807)
Interm. Pr-OS	-	-	-	-	2.27 (0.82-6.26)	0.1121	
Low Pr-OS	-	-	-	-	3.28 (1.26-8.77)	**0.0181***	
**Transcriptomics groups**							
B_{RNA} (ref)	-	-	-	-	*reference*	-	
A_{RNA}	-	-	-	-	2.20 (0.92-5.27)	0.0780	
**HSCAE groups**							
B_{HSCAE} (ref.)	-	-	-	-	*reference*	-	
A_{HSCAE}	-	-	-	-	2.69 (1.01-7.14)	**0.0477***	

NOTE.- Abbreviations: c-index: concordance index, CI: confidence interval, h-RF: handcrafted radiomics features, Interm.: intermediate, HR: hazard ratio, HSCAE: half supervised convolutional autoencoder neural network, MFS: metastatic relapse free survival, No.: number, ref.: reference, Pr-OS: predicted overall survival according to the SARCULATOR nomogram.
*: *P*<0.05, ***: *P*<0.001. Significant results are in bold.

Supplementary Table ST4. Differential gene expression analysis in the $A_{RNA} \times A_{HSCAE}$ group against others.
https://1drv.ms/x/s!ApfK3o5SRuyLgtBXMUolYLB3od9IbQ?e=1ITaoG

Supplementary Table ST5. Geneset analysis in the $A_{RNA} \times A_{HSCAE}$ group against others.
https://1drv.ms/x/s!ApfK3o5SRuyLgtBYA6hZIOVw_RKPwg?e=iTveIS

Supplementary Table ST6. PAMR discriminant analysis in the $A_{RNA} \times A_{HSCAE}$ group against others.
https://1drv.ms/x/s!ApfK3o5SRuyLgtBZMTa6lXY17P6o-Q?e=tG7bYY

Supplementary Table ST7. Bibliography of the genes involved in significantly enriched genesets in the $A_{RNA} \times A_{HSCAE}$ group against others.
https://1drv.ms/x/s!ApfK3o5SRuyLgtEffoLzqV7Tc8jNrw

Supplementary Table ST8. Gene and Pathway enrichment analysis between the CINSARC gene signature and the DGE list of genes identified in our study.

We wanted to determine the intersection between the list of genes of Cinsarc and the list of gene identified by the DGE analysis in our study.

We found that when both Fold Change ≥ 2 and adjusted P-value of 0.05 are enforced, 25/67 (37%) CINSARC genes were found to be significantly expressed in the differential gene expression analysis of the $A_{RNA} \times A_{HSCAE}$ versus Other groups.
In addition, when we enforced only the P-value significance constraint but not the fold change, 54/67 (80%) CINSARC genes have their reproducibility confirmed in the RnaSeq experiment.

Symbol	*CINSARC gene description*	*GeneID*	*P-value Limma t-test*	*Adjusted P-value Limma t-test*	*Lin Fold-change Limma t-test*	*DGE STAT*
MELK	maternal embryonic leucine zipper kinase	9833	5,98076E-05	0,001	-3,65	DOWN
KIF4A	kinesin family member 4A	24137	0,002305012	0,017	-2,93	DOWN
CEP55	centrosomal protein 55kDa	55165	4,24665E-06	0,000	-2,78	DOWN
ANLN	anillin, actin binding protein	54443	2,33616E-05	0,001	-2,69	DOWN
AURKB	aurora kinase B	9212	0,000571305	0,006	-2,56	DOWN
KIF2C	kinesin family member 2C	11004	0,000568411	0,006	-2,54	DOWN
NUF2	NUF2, NDC80 kinetochore complex component	83540	0,000286742	0,004	-2,37	DOWN
TTK	TTK protein kinase	7272	0,005856725	0,032	-2,34	DOWN
CCNB2	cyclin B2	9133	0,004437394	0,027	-2,33	DOWN
CENPA	centromere protein A	1058	2,9803E-05	0,001	-2,31	DOWN
TPX2	TPX2, microtubule-associated	22974	0,000569208	0,006	-2,30	DOWN
PTTG1	pituitary tumor-transforming 1	9232	0,002148233	0,016	-2,20	DOWN
BIRC5	baculoviral IAP repeat containing 5	332	0,004339143	0,026	-2,17	DOWN
TOP2A	topoisomerase (DNA) II alpha 170kDa	7153	0,004854002	0,028	-2,17	DOWN
KIF20A	kinesin family member 20A	10112	0,001460666	0,012	-2,16	DOWN
CENPE	centromere protein E, 312kDa	1062	0,001084569	0,010	-2,13	DOWN
PBK	PDZ binding kinase	55872	0,000399319	0,005	-2,13	DOWN
ASPM	asp (abnormal spindle) homolog, microcephaly associated (Drosophila)	259266	0,001661328	0,013	-2,11	DOWN
KIF23	kinesin family member 23	9493	0,007600857	0,039	-2,09	DOWN
KIF18A	kinesin family member 18A	81930	0,002575121	0,018	-2,07	DOWN
BUB1	BUB1 mitotic checkpoint serine/threonine kinase	699	0,006916652	0,037	-2,05	DOWN
BUB1B	BUB1 mitotic checkpoint serine/threonine kinase B	701	0,003007043	0,020	-2,03	DOWN
CDC20	cell division cycle 20	991	0,009177102	0,045	-2,03	DOWN
CDCA8	cell division cycle associated 8	55143	0,003131918	0,021	-2,00	DOWN
CDCA2	cell division cycle associated 2	157313	0,006072986	0,033	-2,00	EQ
CCNB1	cyclin B1	891	0,002657746	0,019	-1,98	EQ
FOXM1	forkhead box M1	2305	0,004924127	0,029	-1,97	EQ
CDC45	cell division cycle 45	8318	0,00695522	0,037	-1,97	EQ
CKS2	CDC28 protein kinase regulatory subunit 2	1164	0,003070866	0,021	-1,92	EQ
RRM2	ribonucleotide reductase M2	6241	0,001783441	0,014	-1,91	EQ
AURKA	aurora kinase A	6790	0,001090254	0,010	-1,86	EQ
CDK1	cyclin-dependent kinase 1	983	0,004648722	0,028	-1,86	EQ
CDC7	cell division cycle 7	8317	0,007170523	0,038	-1,84	EQ
KIFC1	kinesin family member C1	3833	0,017290559	0,070	-1,83	EQ
KIF14	kinesin family member 14	9928	0,005945762	0,033	-1,79	EQ
CDCA3	cell division cycle associated 3	83461	0,018221564	0,073	-1,77	EQ
PRC1	protein regulator of cytokinesis 1	9055	0,00341883	0,022	-1,75	EQ
ESPL1	extra spindle pole bodies homolog 1 (S. cerevisiae)	9700	0,011287412	0,052	-1,75	EQ
RAD51AP1	RAD51 associated protein 1	10635	0,004233224	0,026	-1,74	EQ
NCAPH	non-SMC condensin I complex, subunit H	23397	0,007334405	0,038	-1,74	EQ
TRIP13	thyroid hormone receptor interactor 13	9319	0,000968955	0,009	-1,73	EQ
OIP5	Opa interacting protein 5	11339	0,009261529	0,045	-1,73	EQ
PLK4	polo-like kinase 4	10733	0,015215254	0,064	-1,69	EQ
CCNA2	cyclin A2	890	0,006745966	0,036	-1,68	EQ
CDC6	cell division cycle 6	990	0,002918138	0,020	-1,67	EQ
FBXO5	F-box protein 5	26271	0,001425341	0,012	-1,64	EQ
ECT2	epithelial cell transforming sequence 2 oncogene	1894	0,001782452	0,014	-1,63	EQ
ZWINT	ZW10 interacting kinetochore protein	11130	0,00992006	0,047	-1,63	EQ
FANCI	Fanconi anemia, complementation group I	55215	0,009010177	0,044	-1,63	EQ
KIF11	kinesin family member 11	3832	0,005268675	0,030	-1,62	EQ

RNASEH2A	ribonuclease H2, subunit A	10535	0,000707178	0,007	-1,60	EQ
SGOL2	shugoshin-like 2 (S. pombe)	151246	0,004499919	0,027	-1,45	EQ
CENPL	centromere protein L	91687	0,00166664	0,013	-1,44	EQ
MAD2L1	MAD2 mitotic arrest deficient-like 1 (yeast)	4085	0,057959585	0,164	-1,41	EQ
MCM2	minichromosome maintenance complex component 2	4171	0,036470738	0,117	-1,41	EQ
KIF15	kinesin family member 15	56992	0,071947331	0,190	-1,39	EQ
CHEK1	checkpoint kinase 1	1111	0,057499924	0,163	-1,39	EQ
SPAG5	sperm associated antigen 5	10615	0,124574679	0,279	-1,37	EQ
SMC2	structural maintenance of chromosomes 2	10592	0,023230237	0,085	-1,34	EQ
NDE1	nudE neurodevelopment protein 1	54820	0,00043775	0,005	-1,34	EQ
NEK2	NIMA-related kinase 2	4751	0,098991786	0,238	-1,31	EQ
BORA	bora, aurora kinase A activator	79866	0,014186587	0,061	-1,30	EQ
H2AFX	H2A histone family, member X	3014	0,174557591	0,352	-1,22	EQ
HP1BP3	heterochromatin protein 1, binding protein 3	50809	0,07348489	0,193	-1,14	EQ
MCM7	minichromosome maintenance complex component 7	4176	0,945786732	0,983	1,04	EQ
SPC25	SPC25, NDC80 kinetochore complex component	57405	0,009623483	0,046	1,33	EQ
PAK3	p21 protein (Cdc42/Rac)-activated kinase 3	5063	0,003483902	0,022	2,00	UP

Also, the CPDB algorithm[41],was used to generate pathway enrichment on both the list of 67 genes of CINSARC[22], and the list of 1230 DGE genes found in the comparison between the $A_{RNA} \times A_{HSCAE}$ versus 'Other' groups of our study.

Seventeen pathways were found in common between the two analyses covering molecular biology functionalities of mitosis control and chromosome integrity. In the original study by Chibon et al.[22], the authors came to similar conclusion concerning the biological interpretation of the CINSARC signature.

Overall, this analysis emphasizes that the DGE analysis between the $A_{RNA} \times A_{HSCAE}$ versus the Other groups is coherent with the genes and functionalities of the CINSARC signature.

Common pathways in the CPDB algorithm	source_DB	source_id	qvalue_Arna_x_Ahscae
Resolution of Sister Chromatid Cohesion	Reactome	R-HSA-2500257	0,00130254
Mitotic Metaphase and Anaphase	Reactome	R-HSA-2555396	0,018701974
Mitotic Anaphase	Reactome	R-HSA-68882	0,017942748
Separation of Sister Chromatids	Reactome	R-HSA-2467813	0,003456507
Amplification of signal from unattached kinetochores via a MAD2 inhibitory signal	Reactome	R-HSA-141444	0,002143352
Amplification of signal from the kinetochores	Reactome	R-HSA-141424	0,002143352
EML4 and NUDC in mitotic spindle formation	Reactome	R-HSA-9648025	0,003119649
Mitotic Spindle Checkpoint	Reactome	R-HSA-69618	0,004878412
RHO GTPases Activate Formins	Reactome	R-HSA-5663220	0,010224915
PLK1 signaling events	PID	plk1_pathway	0,018701974
RHO GTPase Effectors	Reactome	R-HSA-195258	0,029895713
Aurora B signaling	PID	aurora_b_pathway	0,003305051
Regulation of sister chromatid separation at the metaphase-anaphase transition	Wikipathways	WP4240	0,021222033
Kinesins	Reactome	R-HSA-983189	0,045508726
Human T-cell leukemia virus 1 infection - Homo sapiens (human)	KEGG	path:hsa05166	0,005155061
Signal Transduction	Reactome	R-HSA-162582	1,54526E-06
Hemostasis	Reactome	R-HSA-109582	0,003305051

Supplementary Table ST9. Reproducible handcrafted radiomics features selected for the statistical analyses

MRI sequence	Reproducible Handcrafted Radiomics Features	ICC (95% CI)	*P*-value
T2-WI	GLZLM_LZLGE	1 (1 - 1)	<0.001***
	GLRLM_LRE	1 (1 - 1)	<0.001***
	GLRLM_RP	1 (1 - 1)	<0.001***
	GLRLM_SRE	1 (1 - 1)	<0.001***
	GLRLM_GLNU	1 (0.999 - 1)	<0.001***
	GLZLM_LZE	1 (0.999 - 1)	<0.001***
	GLCM_Homogeneity_d1	1 (0.999 - 1)	<0.001***
	GLCM_Homogeneity_d4	1 (0.999 - 1)	<0.001***
	GLRLM_LRHGE	1 (0.999 - 1)	<0.001***
	GLRLM_HGRE	1 (0.999 - 1)	<0.001***
	GLRLM_SRHGE	1 (0.999 - 1)	<0.001***
	GLZLM_GLNU	1 (0.999 - 1)	<0.001***
	GLZLM_LGZE	1 (0.999 - 1)	<0.001***
	GLZLM_ZP	1 (0.999 - 1)	<0.001***
	GLZLM_HGZE	0.999 (0.999 - 1)	<0.001***
	GLCM_Energy_d1	0.999 (0.999 - 1)	<0.001***
	GLCM_Entropy_log10_d4	0.999 (0.999 - 1)	<0.001***
	GLCM_Entropy_log2_d4	0.999 (0.999 - 1)	<0.001***
	GLRLM_RLNU	0.999 (0.998 - 1)	<0.001***
	GLZLM_SZHGE	0.999 (0.999 - 1)	<0.001***
	GLCM_Entropy_log10_d1	0.999 (0.998 - 1)	<0.001***
	GLCM_Entropy_log2_d1	0.999 (0.998 - 1)	<0.001***
	GLZLM_LZHGE	0.999 (0.999 - 1)	<0.001***
	GLZLM_SZLGE	0.999 (0.999 - 1)	<0.001***
	GLCM_Energy_d4	0.999 (0.998 - 1)	<0.001***
	HISTO_Energy	0.999 (0.998 - 1)	<0.001***
	GLZLM_ZLNU	0.999 (0.996 - 1)	<0.001***
	HISTO_Entropy_log2	0.999 (0.997 - 1)	<0.001***
	HISTO_Entropy_log10	0.999 (0.997 - 1)	<0.001***
	NGLDM_Coarseness	0.999 (0.997 - 0.999)	<0.001***
	GLCM_Dissimilarity_d4	0.999 (0.997 - 0.999)	<0.001***
	GLCM_Dissimilarity_d1	0.998 (0.995 - 0.999)	<0.001***
	GLRLM_LRLGE	0.998 (0.996 - 0.999)	<0.001***
	NGLDM_Contrast	0.998 (0.996 - 0.999)	<0.001***
	GLCM_Correlation_d4	0.998 (0.996 - 0.999)	<0.001***
	GLCM_Correlation_d1	0.998 (0.996 - 0.999)	<0.001***
	GLCM_Contrast_d4	0.998 (0.995 - 0.999)	<0.001***
	GLRLM_LGRE	0.997 (0.995 - 0.999)	<0.001***
	GLRLM_SRLGE	0.997 (0.994 - 0.999)	<0.001***
	GLCM_Contrast_d1	0.997 (0.992 - 0.999)	<0.001***
	HISTO_Skewness	0.997 (0.994 - 0.999)	<0.001***
	GLZLM_SZE	0.996 (0.991 - 0.998)	<0.001***
	GLCM_Entropy_log10_d2	0.984 (0.966 - 0.992)	<0.001***
	GLCM_Entropy_log2_d2	0.984 (0.966 - 0.992)	<0.001***
	GLCM_Contrast_d2	0.98 (0.958 - 0.99)	<0.001***
	GLCM_Correlation_d2	0.979 (0.956 - 0.99)	<0.001***
	GLCM_Dissimilarity_d2	0.975 (0.947 - 0.988)	<0.001***
	HISTO_ExcessKurtosis	0.97 (0.936 - 0.986)	<0.001***
	HISTO_Kurtosis	0.97 (0.936 - 0.986)	<0.001***
	GLCM_Energy_d2	0.949 (0.893 - 0.976)	<0.001***
	GLCM_Homogeneity_d2	0.931 (0.855 - 0.967)	<0.001***
FS CE-T1-WI	SHAPE_Compacity	0.999 (0.997 - 0.999)	<0.001***

	SHAPE_Sphericity	0.993 (0.986 - 0.997)	<0.001***
	GLZLM_LZLGE	1 (1 - 1)	<0.001***
	GLRLM_GLNU	1 (1 - 1)	<0.001***
	GLRLM_LRLGE	1 (1 - 1)	<0.001***
	GLRLM_LRE	1 (0.999 - 1)	<0.001***
	GLRLM_RP	1 (0.999 - 1)	<0.001***
	GLZLM_GLNU	1 (0.999 - 1)	<0.001***
	GLCM_Energy_d4	1 (0.999 - 1)	<0.001***
	GLRLM_LGRE	1 (0.999 - 1)	<0.001***
	GLRLM_SRE	1 (0.999 - 1)	<0.001***
	GLRLM_SRLGE	1 (0.999 - 1)	<0.001***
	GLZLM_LZE	1 (0.999 - 1)	<0.001***
	GLRLM_RLNU	1 (0.999 - 1)	<0.001***
	GLZLM_LZHGE	0.999 (0.999 - 1)	<0.001***
	GLZLM_ZP	0.999 (0.999 - 1)	<0.001***
	GLZLM_SZLGE	0.999 (0.999 - 1)	<0.001***
	GLCM_Homogeneity_d1	0.999 (0.997 - 1)	<0.001***
	GLRLM_SRHGE	0.999 (0.998 - 1)	<0.001***
	GLZLM_LGZE	0.999 (0.998 - 1)	<0.001***
	GLRLM_HGRE	0.999 (0.998 - 1)	<0.001***
	GLRLM_LRHGE	0.999 (0.998 - 1)	<0.001***
	GLZLM_SZHGE	0.999 (0.998 - 1)	<0.001***
	GLCM_Homogeneity_d2	0.999 (0.997 - 1)	<0.001***
	GLZLM_HGZE	0.999 (0.998 - 0.999)	<0.001***
	GLCM_Energy_d1	0.999 (0.996 - 0.999)	<0.001***
	GLCM_Entropy_log2_d2	0.999 (0.992 - 1)	<0.001***
	GLCM_Entropy_log10_d2	0.999 (0.992 - 1)	<0.001***
	NGLDM_Coarseness	0.999 (0.996 - 0.999)	<0.001***
	GLCM_Entropy_log10_d1	0.999 (0.99 - 1)	<0.001***
	GLCM_Entropy_log2_d1	0.999 (0.99 - 1)	<0.001***
	HISTO_Kurtosis	0.998 (0.997 - 0.999)	<0.001***
	HISTO_ExcessKurtosis	0.998 (0.997 - 0.999)	<0.001***
	HISTO_Energy	0.998 (0.993 - 0.999)	<0.001***
	NGLDM_Contrast	0.998 (0.996 - 0.999)	<0.001***
	GLCM_Energy_d2	0.998 (0.994 - 0.999)	<0.001***
	GLZLM_ZLNU	0.998 (0.995 - 0.999)	<0.001***
	GLZLM_SZE	0.998 (0.994 - 0.999)	<0.001***
	HISTO_Entropy_log2	0.998 (0.992 - 0.999)	<0.001***
	HISTO_Entropy_log10	0.998 (0.992 - 0.999)	<0.001***
	HISTO_Skewness	0.997 (0.994 - 0.999)	<0.001***
	GLCM_Dissimilarity_d1	0.997 (0.993 - 0.999)	<0.001***
	GLCM_Dissimilarity_d2	0.997 (0.993 - 0.998)	<0.001***
	GLCM_Correlation_d1	0.996 (0.992 - 0.998)	<0.001***
	GLCM_Contrast_d1	0.996 (0.991 - 0.998)	<0.001***
	GLCM_Correlation_d2	0.995 (0.99 - 0.998)	<0.001***
	NGLDM_Busyness	0.995 (0.989 - 0.997)	<0.001***
	GLCM_Contrast_d2	0.995 (0.989 - 0.997)	<0.001***
	GLCM_Contrast_d4	0.994 (0.988 - 0.997)	<0.001***
	GLCM_Dissimilarity_d4	0.988 (0.975 - 0.995)	<0.001***
	GLCM_Entropy_log10_d4	0.982 (0.961 - 0.991)	<0.001***
	GLCM_Entropy_log2_d4	0.982 (0.961 - 0.991)	<0.001***
	GLCM_Correlation_d4	0.975 (0.946 - 0.988)	<0.001***
	GLCM_Homogeneity_d4	0.971 (0.938 - 0.986)	<0.001***
T1-WI	GLCM_Homogeneity_d2	0.999 (0.998 - 1)	<0.001***
	GLCM_Homogeneity_d4	0.999 (0.998 - 1)	<0.001***
	GLCM_Entropy_log2_d2	0.999 (0.996 - 1)	<0.001***

GLCM_Entropy_log10_d2	0.999 (0.996 - 1)	<0.001***
GLCM_Correlation_d4	0.999 (0.998 - 1)	<0.001***
GLCM_Entropy_log10_d4	0.999 (0.996 - 1)	<0.001***
GLCM_Entropy_log2_d4	0.999 (0.996 - 1)	<0.001***
GLZLM_LZHGE	0.999 (0.998 - 1)	<0.001***
GLRLM_LRE	0.999 (0.997 - 0.999)	<0.001***
GLCM_Correlation_d2	0.998 (0.996 - 0.999)	<0.001***
GLCM_Energy_d2	0.998 (0.995 - 0.999)	<0.001***
GLCM_Dissimilarity_d4	0.998 (0.993 - 0.999)	<0.001***
GLCM_Energy_d4	0.998 (0.995 - 0.999)	<0.001***
GLCM_Energy_d1	0.997 (0.994 - 0.999)	<0.001***
GLCM_Dissimilarity_d2	0.997 (0.991 - 0.999)	<0.001***
GLRLM_RP	0.994 (0.987 - 0.997)	<0.001***
GLRLM_LRHGE	0.992 (0.983 - 0.996)	<0.001***
GLCM_Contrast_d4	0.992 (0.976 - 0.996)	<0.001***
GLCM_Correlation_d1	0.99 (0.978 - 0.995)	<0.001***
HISTO_Energy	0.99 (0.978 - 0.995)	<0.001***
GLRLM_SRE	0.988 (0.974 - 0.994)	<0.001***
GLCM_Contrast_d2	0.988 (0.968 - 0.995)	<0.001***
NGLDM_Coarseness	0.986 (0.97 - 0.993)	<0.001***
GLCM_Entropy_log10_d1	0.986 (0.969 - 0.993)	<0.001***
GLCM_Entropy_log2_d1	0.986 (0.969 - 0.993)	<0.001***
GLRLM_LRLGE	0.983 (0.965 - 0.992)	<0.001***
GLCM_Homogeneity_d1	0.983 (0.963 - 0.992)	<0.001***
GLZLM_ZLNU	0.978 (0.954 - 0.99)	<0.001***
GLZLM_LZE	0.975 (0.946 - 0.988)	<0.001***
HISTO_Entropy_log10	0.97 (0.935 - 0.986)	<0.001***
HISTO_Entropy_log2	0.97 (0.935 - 0.986)	<0.001***
GLZLM_SZLGE	0.964 (0.925 - 0.983)	<0.001***
GLZLM_SZE	0.963 (0.917 - 0.983)	<0.001***
GLZLM_LGZE	0.956 (0.906 - 0.979)	<0.001***
GLZLM_LZLGE	0.956 (0.905 - 0.979)	<0.001***
GLRLM_HGRE	0.954 (0.903 - 0.978)	<0.001***
HISTO_Skewness	0.951 (0.894 - 0.977)	<0.001***
GLRLM_SRHGE	0.951 (0.896 - 0.977)	<0.001***
GLZLM_HGZE	0.947 (0.888 - 0.975)	<0.001***
GLRLM_LGRE	0.944 (0.883 - 0.974)	<0.001***
GLRLM_SRLGE	0.941 (0.876 - 0.972)	<0.001***
GLZLM_GLNU	0.925 (0.842 - 0.965)	<0.001***
GLCM_Dissimilarity_d1	0.924 (0.837 - 0.964)	<0.001***
NGLDM_Contrast	0.923 (0.837 - 0.964)	<0.001***
GLRLM_RLNU	0.923 (0.838 - 0.964)	<0.001***
GLZLM_SZHGE	0.919 (0.828 - 0.962)	<0.001***

NOTE.- Tests are intra-class correlation coefficient (ICC, with 95% confidence interval [CI]) with the following arguments: two-way model, agreement type, average unit.
***: $P<0.001$

Supplementary Table ST10. Open-access software, packages and functions used in the analyses of the study.

Softwares and packages	Source	Version	Functions
Conda environment	https://docs.anaconda.com/	v4.8	Full list of packages included in the environments is available on request
R	https://www.r-project.org/	v3.6.3 and v4.1.0	
Rstudio	http://www.rstudio.com/products/rstudio/	v1.4.1106	
R Bioconductor (BiocInstaller)	https://www.bioconductor.org	V1.20.3	-
Keras	https://github.com/fchollet/keras	v2.3.0.0	-
**MRI post-processing**			
"ANTsR" R package	https://github.com/ANTsX/ANTsR	v0.5.6.0.0	
"ANTsRCore" R package	https://github.com/ANTsX/ANTsRCore	v0.7.4	denoiseImage, antsImageRead, antsImageWrite
"oro.dicom" R package	https://github.com/cran/oro.dicom	v0.5.3	readDICOM, dicom2nifti
"oro.nifti" R package	https://github.com/neuroconductor/oro.nifti	v0.11.0	writeNIfTI
"extrantsr" R package	https://github.com/muschellij2/extrantsr	v3.9.1	resample_image
"irr" R package	https://github.com/cran/irr	v0.84.1	icc
**Statistical analyses and data manipulation**			
"survival" R package	https://github.com/therneau/survival	v3.3-3<br>v3.2-11	coxph, Surv, survdiff, survfit, summary, predict
"stats" R package	https://github.com/SurajGupta/r-source/blob/master/src/library/stats/R/	v4.1.0	chisq.test, table, shapiro.test, cor.test, wilcox.test, t.test, hclust
"rms" R package	https://github.com/cran/rms	v6.2-0	cph, validate
"base" R package	https://github.com/SurajGupta/r-source/blob/master/src/library/base/R/	v4.1.0	table, min, max, sd, quantile, summary, hclust, p.adjust
"MASS" R package	https://github.com/cran/MASS	v7.3-54	stepAIC
"boot" R package	https://github.com/cran/boot	v1.3-28	boot, boot.ci
"dplyr" R package	https://github.com/tidyverse/dplyr	v1.1.2	filter, select, arrange, summarize
"purrr" R package	https://github.com/tidyverse/purrr	v0.3.4	map, map2, safely
"'ConsensusClusterPlus" R package	https://github.com/renzhonglu/ConsensusClusterPlus/blob/master/R/ConsensusClusterPlus.R	v1.24.0	ConsensusClusterPlus
"caret" R package	https://github.com/topepo/caret/	v6.0.86	createDataPartition
"sva" Bioconductor package	https://www.bioconductor.org/packages/release/bioc/html/sva.html		ComBat
"limma" Bioconductor package	https://bioconductor.org/packages/release/bioc/html/limma.html	v3.26.9	voom
"pamr" R package	https://cran.r-project.org/web/packages/pamr/	v1.55	
**Data visualization**			
"survminer" R package	https://github.com/kassambara/survminer	v0.4.9	ggsurvplot
"ggplot2" R package	https://github.com/tidyverse/ggplot2	v3.4.2	ggplot, ggsave
"cowplot" R package	https://github.com/wilkelab/cowplot	v1.1.1	plot_grid
"magick" R package	https://imagemagick.org/	v2.4.0	image_flip<br>image_flop<br>image_shear<br>image_rotate<br>image_extent
**Bioinformatics**			
Fastq Paired End Validator	https://github.com/orionzhou/luffy/blob/master/archive/FastqPairedEndValidator.pl	v0.1.0	
BBmap	https://github.com/BioInfoTools/BBMap	v37.57	clumpify
Sickle	https://github.com/najoshi/sickle	v1.33	

SeqPrep	https://github.com/jstjohn/SeqPrep	v0.0.1	
Bowtie2	https://github.com/BenLangmead/bowtie2	v2.1.0	
TopHat2	http://ccb.jhu.edu/software/tophat/index.shtml	v2.0.10	
PicardTools	https://broadinstitute.github.io/picard/	v1.1.118	MarkDuplicates
UCSC Hg19 reference genome	https://hgdownload2.soe.ucsc.edu/goldenPath/hg19/chromosomes/<br>https://www.ncbi.nlm.nih.gov/datasets/genome/GCF_000001405.13/		
UCSC Hg19 reference transcriptome	RefSeq coding table was generated using UCSC table browser :<br>https://genome.ucsc.edu/cgi-bin/hgTables?hgsid=2016435530_b8tYp3iYUs135gCWAFwoCcVylHe9&clade=mammal&org=Human&db=hg19&hgta_group=genes&hgta_track=refSeqComposite&hgta_table=0&hgta_regionType=genome&position=chr1%3A3%2C049%2C860-3%2C105%2C960&hgta_outputType=gff&hgta_outFileName=refseq_hg19		

Supplementary Method M1. Deep Learning methodology
Deep Learning Methodology and techniques are described in the annex file: https://1drv.ms/w/s!ApfK3o5SRuyLgtgbKDtdJGFsT7V-Cg?e=6T93VU

*

Supplementary Method M2. RNA-sequencing.

The custom code used to generate the results presented in this paper is available from the corresponding author upon reasonable request.

The transcriptomics analysis was conducted in all patients with pre-treatment frozen and paraffin-embedded samples. After whole-RNA sequencing, the produced RNA sequences were quality controlled and aligned to the transcriptome. Gene Expression was then estimated by the counts of high-quality sequences aligned per Gene. Finally gene expression counts were normalized using the Voom method[32].

Sequencing protocol.
Regarding the 54 patients with frozen samples: RNA extraction from frozen tissue was performed at Institut Bergonié using the Rneasy mini Kit from Qiagen (Qiagen, Venlo, Netherlands). Quality control of RNA samples were: (1) Nanodrop: Preliminary quantitation and purity samples assessment, (2) Qubit 2.0 : Sensitive quantitation. NGS library preparation and sequencing were performed by Integragen (Integragen, Evry, France). NGS library preparation was done using NEBNext Ultra II mRNA-Seq Kit from NEB (New England Biolabs, Ipswich, Massachusetts, United States). RNA-Sequencing was performed on NovaSeq 6000 platform (illumina Inc. San Diego, Ca, USA) via the library described below. The qualified libraries were loaded into Illumina sequencers after pooling according to their effective concentrations and expected data volumes. Paired-end RNA sequencing was performed with paired-end sequences of length 100x2 nc. Target number of RNA sequences pairs was 65M per patient.
Regarding the 56 patients with FFPE samples: RNA extraction from FFPE samples was performed at Institut Bergonié using a Maxwell RSC RNA FFPE Kit AS1440 (Promega Corporation, Madison, Wisconsin, United States). Quality control of RNA samples were: (1) Nanodrop: Preliminary quantitation, (2) Agilent 2100: checks RNA integrity and quantitation. NGS library preparation and sequencing were performed by Novogene (Novogene Corporation, China). NGS library preparation was done using directional RNA library (rRNA removal). NGS sequencing was done as follows. RNA-Sequencing was performed on NovaSeq 6000 platform (Illumina Inc. San Diego, Ca, USA) The qualified libraries were loaded into Illumina sequencers after pooling according to its effective concentration and expected data volume. Paired-end sequencing was performed with paired-end sequences of length 150x2 nc. Target number of RNA sequences of 60M per patient.

Sequencing Alignement.
Starting from the sequences produce by Rna Seq Whole Transcriptome sequencing, bioinformatics analysis was performed based on the Hg19/Gh37 version of the human genome.
Briefly, pre-alignment and quality control of sequences (.fastQ.gz) was done via FastqPairedEndValidator to check that R1 and R2 sequences were correctly paired (https://github.com/orionzhou/luffy/blob/master/archive/FastqPairedEndValidator.pl), Clumpify to remove reads having identical sequence (https://github.com/BioInfoTools/BBMap) and Sickle to trim low quality sequences at 5' and

3’ ends (Sickle - https://github.com/najoshi/sickle). SeqPrep package (SeqPrep - https://github.com/jstjohn/SeqPrep) was used to remove sequencing adaptors from raw reads. This package also detected the proportion of nucleotide fragments whose R1 and R2 paired end reads were overlapping and merged them into single-end reads.
To prevent double coverage bias due to overlapping R1 and R2 sequences and keep exploiting those fragments, a home-made python script was developed to split those merged reads into new non-overlapping R1 and R2 paired-end reads. Alignment of quality controlled DNA sequences to .bam files was performed via Bowtie2 (1) with the ‘–very-sensitive’ alignment strategy parameter. Alignment of RNA sequences was performed using Tophat2 (2) and Bowtie2 (1) on both the UCSC hg19 reference genome and transcriptome. Remaining PCR duplicates reads in Post-alignment .bam files were removed via the PicardTools suite MarkDuplicates module (PicardTools - https://broadinstitute.github.io/picard/).

Normalization of Gene Expression data.
ComBat harmonization method was applied to correct for batch effect[31] between the 54 patients with frozen samples and the 56 patients with FFPE samples. We used the Voom method to normalize the trancript counts of our samples. This is a method that transforms count data to log2-counts per million (logCPM), then estimates the mean-variance relationship and finally uses this relationship to compute appropriate observationnal-level weights.

*

Supplementary Method M3. Gene-expression profiles of the radiomics groups.

The custom code used to generate the results presented in this paper is available from the corresponding author upon reasonable request.

Differential gene expression (DGE) and Geneset enrichment analyses between the radiomics groups determined by unsupervised clustering were performed as follows.

Differential analysis.
The RNA-Seq differential gene expression between defined groups was performed using the statistical t-test from the ‘LIMMA’ R package. It calculates fold change and nominal P-values related to each gene starting from raw expression values and the normalization weights produced by the ‘VOOM’ R package. The set of nominal P-values were adjusted using Benjamini-Hochberg procedure. The P-value threshold cut-off to discriminate significant up/down regulated transcripts was set to 0.05 and the fold change was set to 2.0[40].

Geneset enrichment analysis.
We assessed enrichment in biological pathways between RNA-Sequencing groups based on Broad Institute’s Molecular Signature Data Base gene sets (MSigDB) and the CYBERSORT LM22 immuno gene sets. LM22 is a signature matrix file consisting of 547 genes that accurately distinguish 22 mature human hematopoietic populations isolated from peripheral blood or *in vitro* culture conditions, including seven T cell types, naïve and memory B cells, plasma cells, NK cells, and myeloid subsets[33,34]. Gene-set enrichment was performed as follows: the total count of genes (UNIVERSE) was set to the total number of genes for which the Differential Gene Expression (DGE) calculation was performed (18,399 annotated genes in our study). For each biological pathway we counted the genes that participated in the pathway (IN.GENESET) and its complement with respect to the UNIVERSE (NOT.IN.GENESET). Also the UNIVERSE was split in differentially expressed genes (SIG.DGE) and their complement (NOT.SIG.DGE). Then we built a 2 x 2 contingency matrix holding the count of differentially expressed genes in the pathway (SIG.DGE.IN.GENESET) and its complement (SIG.DGE.NOT.IN.GENESET) and the count of not differentially expressed genes in the pathway (NOT.SIG.DGE.IN.GENESET) and its complement

(NOT.SIG.DGE.NOT.IN.GENESET). We performed the hypergeometric test on this table via fisher test in R.
The raw P-values of all tests for each pathway were adjusted. We additionally performed the calculation of the odds ratio (OR) based on the above contingency table and its 95% confidence interval (CI, results of the fisher.test function R base package). We tagged as significantly ENRICHED all pathways with a significant hypergeometric test at adjusted P-value ≤ 0.05 and the lower bound of the 95%CI of their OR not lower than 1. We ranked all ENRICHED-tagged pathways by the count of differentially expressed genes in the pathway (SIG.DGE.IN.GENSET). Then we wanted to qualitatively assess pathways significantly over-expressed or under-expressed in sample groups. To that extent, and for each pathway and each group, we calculate the ratio between the numbers of genes that were over-expressed in the pathway and the numbers of DGE genes in the pathway. A ratio close to 0 suggests that the pathway was under-expressed while a ratio close to 1 suggested that the pathway was over-expressed in the reference group. Ratios that were close to 0.5 were considered informative of no enrichment in either group.

PAMr analysis.
Prediction Analysis for Microarrays (PAM) provides a method for class prediction from gene expression profiling, based on an enhancement of the simple nearest prototype (centroid) classifier[35]. The 'nearest shrunken centroids' identifies subsets of genes that best characterize each class. Briefly, we provided to PAMr the full expression matrix normalized as previously described in Supplementary methods. Each sample was assigned to a specific class of interest to determine a subset of discriminant genes for binary classification. The signature was chosen based on PAM shrunken fold change threshold of 2.8 and FDR of 0.05 that guaranteed a mean binary class cross-validation error of 10% (cross-validation by 100 random sample reshuffling iterations)
