# Supplementary material for: Integration of pre-treatment computational radiomics, deep radiomics, and transcriptomics enhances soft-tissue sarcoma patient prognosis
Source: NPJ Precis Oncol. 2024 Jun 7;8:129. doi: 10.1038/s41698-024-00616-8 (PMC11161510; doi:10.1038/s41698-024-00616-8)
Supplement: Supplementary file 2 — Reporting Summary [file 41698_2024_616_MOESM2_ESM.pdf]

nature portfolio

Corresponding author(s): Amandine Crombé

Last updated by author(s): Mar 18, 2024

Reporting Summary

Nature Portfolio wishes to improve the reproducibility of the work that we publish. This form provides structure for consistency and transparency in reporting. For further information on Nature Portfolio policies, see our Editorial Policies and the Editorial Policy Checklist.

Statistics

For all statistical analyses, confirm that the following items are present in the figure legend, table legend, main text, or Methods section.

n/a	Confirmed	
☐	☒	The exact sample size (*n*) for each experimental group/condition, given as a discrete number and unit of measurement
☐	☒	A statement on whether measurements were taken from distinct samples or whether the same sample was measured repeatedly
☐	☒	The statistical test(s) used AND whether they are one- or two-sided<br>*Only common tests should be described solely by name; describe more complex techniques in the Methods section.*
☐	☒	A description of all covariates tested
☐	☒	A description of any assumptions or corrections, such as tests of normality and adjustment for multiple comparisons
☐	☒	A full description of the statistical parameters including central tendency (e.g. means) or other basic estimates (e.g. regression coefficient) AND variation (e.g. standard deviation) or associated estimates of uncertainty (e.g. confidence intervals)
☐	☒	For null hypothesis testing, the test statistic (e.g. *F*, *t*, *r*) with confidence intervals, effect sizes, degrees of freedom and *P* value noted<br>*Give P values as exact values whenever suitable.*
☒	☐	For Bayesian analysis, information on the choice of priors and Markov chain Monte Carlo settings
☒	☐	For hierarchical and complex designs, identification of the appropriate level for tests and full reporting of outcomes
☒	☐	Estimates of effect sizes (e.g. Cohen's *d*, Pearson's *r*), indicating how they were calculated

Our web collection on statistics for biologists contains articles on many of the points above.

Software and code

Policy information about availability of computer code

Data collection	The data collection was performed in the medical files, in the pathological reports and archives and in the picture archiving and communication systems from Bergonié institute.
Data analysis	The underlying code for this study is not publicly available but may be made available to qualified researchers on reasonable request from the corresponding author. The R studio and Conda environments and the packages with their versions used for the analyses are detailed in Supplementary Table ST10.

For manuscripts utilizing custom algorithms or software that are central to the research but not yet described in published literature, software must be made available to editors and reviewers. We strongly encourage code deposition in a community repository (e.g. GitHub). See the Nature Portfolio guidelines for submitting code & software for further information.

Data

Policy information about availability of data

All manuscripts must include a data availability statement. This statement should provide the following information, where applicable:

- Accession codes, unique identifiers, or web links for publicly available datasets
- A description of any restrictions on data availability
- For clinical datasets or third party data, please ensure that the statement adheres to our policy

The raw and processed data generated in this study have been deposited in NCBI's Gene Expression Omnibus (GEO) and are accessible through GEO Series

accession numbers [GEO1-still ongoing] and [GEO2-still ongoing] for RNA-seq experiment. The differential gene expression analyses and pathways analyses are available in the supplementary materials. The radiomics datasets and raw MRIs used and/or analysed during the current study are available from the corresponding author on reasonable request. Any additional results can be obtained from the corresponding author.

Research involving human participants, their data, or biological material

Policy information about studies with human participants or human data. See also policy information about sex, gender (identity/presentation), and sexual orientation and race, ethnicity and racism.

Reporting on sex and gender	Yes,we have carefully reported data about sex and gender.
Reporting on race, ethnicity, or other socially relevant groupings	Not applicable
Population characteristics	Yes, we have carefully described the characteristics of the population, namely: age, sex, performans status, histologic grade, histologic types, tumor size, location and depth, adjuvant treatments and outcomes.
Recruitment	We have described that patients were all consecutive patientes managed at our sarcoma reference center between May 2008 and May 2020
Ethics oversight	Institutional review board from Bergonié institute, comprehensive cancer center of Bordeaux, Nouvelle-Aquitaine, France

Note that full information on the approval of the study protocol must also be provided in the manuscript.

Field-specific reporting

Please select the one below that is the best fit for your research. If you are not sure, read the appropriate sections before making your selection.

☒ Life sciences ☐ Behavioural & social sciences ☐ Ecological, evolutionary & environmental sciences

For a reference copy of the document with all sections, see nature.com/documents/nr-reporting-summary-flat.pdf

Life sciences study design

All studies must disclose on these points even when the disclosure is negative.

Sample size	We included all the patients fulfilling the inclusion criteria. The population size was not calculated but empirical, and larger than any prior study on multi-omics analysis in sarcoma. The largest population ever on radiomics analysis (only) corresponds to 226 patients (Peeken et al., EBioMedicine, 2019) against 225 patients here.
Data exclusions	We excluded patients with atypical lipomatous tumors, metastases at initial staging (i.e., on chest CT-scan), and patients whom pre-treatment MRI did not include at least one T1-weighted imaging (WI), one T2-WI and one fat-suppressed (FS) CE T1-WI (CE-T1-WI).
Replication	Tumor segmentations were performed twice and only reproducible radiomics features were selected for the radiomics modeling. Two senior radiologists verified all the segmentations.
Randomization	not applicable
Blinding	For the semantic radiological analysis and the radiomics analyses, radiologists were blinded from the patients' data

Reporting for specific materials, systems and methods

We require information from authors about some types of materials, experimental systems and methods used in many studies. Here, indicate whether each material, system or method listed is relevant to your study. If you are not sure if a list item applies to your research, read the appropriate section before selecting a response.

Materials & experimental systems

n/a	Involved in the study	
☒	☐	Antibodies
☒	☐	Eukaryotic cell lines
☒	☐	Palaeontology and archaeology
☒	☐	Animals and other organisms
☐	☒	Clinical data
☒	☐	Dual use research of concern
☒	☐	Plants

Methods

n/a	Involved in the study	
☐	☒	ChIP-seq
☒	☐	Flow cytometry
☐	☒	MRI-based neuroimaging

Clinical data

Policy information about clinical studies

All manuscripts should comply with the ICMJE guidelines for publication of clinical research and a completed CONSORT checklist must be included with all submissions.

Clinical trial registration: not applicable

Study protocol: not applicable : this is a retrospective observational study. All the methods is explained in the Materials and Methods section and in supplementary Data.

Data collection: Data collection was achieved using medical files, pathological files, pathological archives and radiological PACS between May 2008 and May 2020

Outcomes: ollow-ups consisted of clinical examinations and chest radiographs every 3 months for 2 years, then every 6 months for 5 years, and then annually, with complementary local MRI, and chest CT-scan in case of doubtful findings. The main outcome was MFS. MFS, local relapse-free survival (LFS) and overall survival (OS) corresponded to the time (in months) elapsed from surgery to metastatic relapse, local relapse and death related to disease (or last follow-up), respectively. Patients without event during the study period were censored. All relapses were histologically proven.

Plants

Seed stocks: not applicable

Novel plant genotypes: not applicable

Authentication: not applicable

ChIP-seq

Data deposition

☒ Confirm that both raw and final processed data have been deposited in a public database such as GEO.

☐ Confirm that you have deposited or provided access to graph files (e.g. BED files) for the called peaks.

Data access links
May remain private before publication.: We have uploaded the raw and porcessed data on GEO and it is still ongoing. The confirmation numbers are uploads/0009-0007-9907-6214@orcid_iNu9eWM6/geo_radiomics_2024_batch2 and uploads/0009-0007-9907-6214@orcid_iNu9eWM6/geo_radiomics_2024_batch1

Files in database submission: The raw and processed data generated in this study have been deposited in NCBI's Gene Expression Omnibus (GEO) and are accessible through GEO Series accession numbers [GEO1/ongoing] and [GEO2/ongoing] for RNA-seq experiment. The differential gene expression analyses and pathways analyses are available in the supplementary materials. The radiomics datasets and raw MRIs used and/or analysed during the current study are available from the corresponding author on reasonable request. Any additional results can be obtained from the corresponding author.

Genome browser session
(e.g. UCSC): the final link is not already available as the data are still loading (1.7 To)

Methodology

Replicates	not applicable
Sequencing depth	Sequencing protocol.<br>Regarding the 54 patients with frozen samples: RNA extraction from frozen tissue was performed at Institut Bergonié using the Rneasy mini Kit from Qiagen (Qiagen, Venlo, Netherlands). Quality control of RNA samples were: (1) Nanodrop: Preliminary quantitation and purity samples assessment, (2) Qubit 2.0 : Sensitive quantitation. NGS library preparation and sequencing were performed by Integragen (Integragen, Evry, France). NGS library preparation was done using NEBNext Ultra II mRNA-Seq Kit from NEB (New England Biolabs, Ipswich, Massachusetts, United States). RNA-Sequencing was performed on NovaSeq 6000 platform (illumina Inc. San Diego, Ca, USA) via the library described below. The qualified libraries were loaded into Illumina sequencers after pooling according to their effective concentrations and expected data volumes. Paired-end RNA sequencing was performed with paired-end sequences of length 100x2 nc. Target number of RNA sequences pairs was 65M per patient.<br>Regarding the 56 patients with FFPE samples: RNA extraction from FFPE samples was performed at Institut Bergonié using a Maxwell RSC RNA FFPE Kit AS1440 (Promega Corporation, Madison, Wisconsin, United States). Quality control of RNA samples were: (1) Nanodrop: Preliminary quantitation, (2) Agilent 2100: checks RNA integrity and quantitation. NGS library preparation and sequencing were performed by Novogene (Novogene Corporation, China). NGS library preparation was done using directional RNA library (rRNA removal). NGS sequencing was done as follows. RNA-Sequencing was performed on NovaSeq 6000 platform (Illumina Inc. San Diego, Ca, USA) The qualified libraries were loaded into Illumina sequencers after pooling according to its effective concentration and expected data volume. Paired-end sequencing was performed with paired-end sequences of length 150x2 nc. Target number of RNA sequences of 60M per patient.<br><br>Sequencing Alignement.<br>Starting from the sequences produce by Rna Seq Whole Transcriptome sequencing, bioinformatics analysis was performed based on the Hg19/Gh37 version of the human genome.<br>Briefly, pre-alignment and quality control of sequences (.fastQ.gz) was done via FastqPairedEndValidator to check that R1 and R2 sequences were correctly paired (https://github.com/orionzhou/luffy/blob/master/archive/FastqPairedEndValidator.pl), Clumpify to remove reads having identical sequence (https://github.com/BioInfoTools/BBMap) and Sickle to trim low quality sequences at 5' and 3' ends (Sickle - https://github.com/najoshi/sickle). SeqPrep package (SeqPrep - https://github.com/jstjohn/SeqPrep) was used to remove sequencing adaptors from raw reads. This package also detected the proportion of nucleotide fragments whose R1 and R2 paired end reads were overlapping and merged them into single-end reads.<br>To prevent double coverage bias due to overlapping R1 and R2 sequences and keep exploiting those fragments, a home-made python script was developed to split those merged reads into new non-overlapping R1 and R2 paired-end reads. Alignment of quality controlled DNA sequences to .bam files was performed via Bowtie2 (1) with the '–very-sensitive' alignment strategy parameter. Alignment of RNA sequences was performed using Tophat2 (2) and Bowtie2 (1) on both the UCSC hg19 reference genome and transcriptome. Remaining PCR duplicates reads in Post-alignment .bam files were removed via the PicardTools suite MarkDuplicates module (PicardTools - https://broadinstitute.github.io/picard/).
Antibodies	not applicable
Peak calling parameters	not applicable
Data quality	Normalization of Gene Expression data.<br>ComBat harmonization method was applied to correct for batch effect31 between the 54 patients with frozen samples and the 56 patients with FFPE samples. We used the Voom method to normalize the trancript counts of our samples. This is a method that transforms count data to log2-counts per million (logCPM), then estimates the mean-variance relationship and finally uses this relationship to compute appropriate observationnal-level weights.
Software	The R packages used for the RNAseq analyses are described in Supplementary Table ST10 : Fastq Paired End Validator, BBmap, Sickle, SeqPrep, Bowtie2, TopHat2, PicardTools, UCSC Hg19 reference genome, UCSC Hg19 reference transcriptome

Magnetic resonance imaging

Experimental design

Design type	not applicable
Design specifications	not applicable
Behavioral performance measures	not applicable

Acquisition

Imaging type(s)	conventional MRI
Field strength	1.5 Tesla
Sequence & imaging parameters	spin echo and gradient echo sequences : T1 weighted imaging, T2 weighted imaging, fat suppressed T1 weighted imaging after gadolinium chelates injection.
Area of acquisition	trunk wall, upper and lower limbs

Diffusion MRI ☐ Used ☒ Not used

Preprocessing

Preprocessing software	LIFEx open source freeware (v4.70.0) and ITK (insight tool kit) open source library.
Normalization	intensity harmonization techniques using the ITK (insight tool kit) open source library and histogram matching , standardization of voxel size with bilinear interpolation (ITK library) ; N4 bias field correction (ITK library) ; gray levels standardization with LIFEx.
Normalization template	intensity harmonization techniques using the ITK (insight tool kit) open source library
Noise and artifact removal	not applicable
Volume censoring	not applicable

Statistical modeling & inference

Model type and settings	Extraction of radiomics features followed by filtering of the reproducible features used in unsupervised clustering
Effect(s) tested	not applicable

Specify type of analysis: ☐ Whole brain ☒ ROI-based ☐ Both

Anatomical location(s): *Describe how anatomical locations were determined (e.g. specify whether automated labeling algorithms or probabilistic atlases were used).*

Statistic type for inference (See Eklund et al. 2016)	not applicable
Correction	not applicable

Models & analysis

n/a	Involved in the study
☒	☐ Functional and/or effective connectivity
☒	☐ Graph analysis
☒	☐ Multivariate modeling or predictive analysis
